# The power of the people: the meaning of *kratos* in *dēmokratia*

**DOI:** 10.12688/openreseurope.13726.1

**Published:** 2021-05-25

**Authors:** John McGuire

**Affiliations:** 1 School of Philosophy, University College Dublin, Belfield, Dublin, Dublin 4, Ireland

**Keywords:** Ancient and Modern Democracy; Democratic power and discontent; Populism; Demosthenes; Aristophanes; Aeschylus; Josiah Ober

## Abstract

In this paper, I reconstruct the notion of *kratos* as a unique and distinguishable exercise of political power. Using examples from 5th- and 4th-century Attic tragedy, Old Comedy, and forensic oratory, I show how *kratos* was used in Athenian cultural and political discourse to convey the *irrefutability* of a claim, the recognition of someone’s *prevailing* over another, and the sense of *having the last word*—all of which makes kratic power dependent upon its own continued demonstrability. I argue that the peculiarly performative character of kratos has little or no role within contemporary democratic thinking because the agency of the dēmos is largely mediated through the mechanisms of electoral success and constitutional rights. Nevertheless—and regardless of whether they are ultimately successful in achieving their stated political aims—the spontaneous, organisationally diffuse protests operating *extra-institutionally *under the banners of #MeToo and Black Lives Matter reveal how the attempted ‘domestication’ of *kratos*, and the sublimation of its peculiar power into piecemeal reform, was never a realistic or satisfactory answer for democratic discontent.

## Introduction

Political theory has tended to treat the resurgence of populist discontent as a symptom of cultural or institutional dysfunction, for which the only remedy is to refine and defend democracy’s fundaments against disfigurement by mass mobilisation ^1^. The sense of exasperation is even less subtle outside the academy:

“People are still out protesting. You don’t need to protest. You won. You accomplished your goal. Society says, you’re right, the police need systemic reform. That was accomplishment one. Now go to step two. What reform do you want?…How do we redesign the police department? We start with this. It’s a blank piece of paper. What do you want the police department to be in New York City? Let’s design it. Here’s a pen.” ^2^
“When some urged us to use force immediately, we chose dialogue and mutual respect. When others urged us to give up, we extended a hand in good faith…The situation as it currently stands is unacceptable and untenable…Our government has been patient. But it has been two weeks, and the barricades need to come down now.” ^3^
“[W]e seem to be breeding a generation of students, mostly female students, deploying Title IX to remedy sexual ambivalences or awkward sexual experiences…If this is what feminism on campus has come to, then seriously, let’s just cash it in and start over, because this feminism is broken.” ^4^

The adamancy and insolence fuelling each new outbreak of mass protest is reliably met with high-handed dismissals of grassroots activists as directionless, excessively militant, and ruinous for ‘real’ democratic politics. Halting the construction of a pipeline, defunding of police, anonymously publishing names of alleged sexual abusers—such extra-institutional initiatives skirt too close to mob rule to be acceptable manifestations of democratic power. But as legitimate political participation becomes narrowcasted to voting (and occasionally canvassing) for one’s preferred candidate, democratic constitutions begin to resemble esoteric *grimoires*, discernible only to a select group of technocrats, pundits, and theorists. It is not the aim of this paper to assess the effectiveness of anti-establishment sentiment, nor to judge whether populism is ultimately compatible with democratic pluralism. Instead, I want to try and recapture some of the basic intuitions underpinning ancient Athenian democracy and the peculiar mode of power expressed as *kratos*, in the hopes of improving its legibility within contemporary political debates ^5^. In pursuing this, I also mean to challenge a prevalent assumption that democracy’s viability as a normative-organisational principle necessitates the taming and domesticating popular discontent.

 For better or worse, Attic coinages continue to serve as anchoring terms in Western political discourse. Indeed, not only the general category of our politics (τὰ πολιτικά), but many of its most contentious labels are phonetic approximations of this same Ionian dialect: αὐτόνομος (autonomous), βάρβαρος (barbarous), δημοκρατία (democracy), δεσπότης (despot), κυνικός (cynical), μανία (mania), ὀλιγαρχία (oligarchy), συκοφάντης (sycophant), τύραννος (tyrant). And while opinion polls continue to suggest widespread popular support for norms linked to democracy (including gender non-discrimination and equality before the law), many remain at a loss to explain how their agency feeds into this system ^6^.

 By contrast, when we consider the workings of ancient democracy (at least as experienced by the privileged class of men who could claim political membership) we imagine a much more direct and appreciable grasp on power. Part of what made ancient democratic power intuitable for ordinary citizens was linguistic: back when Attic Greek held currency, novel political coinages could be decomposed into parts and synecdochally grasped. We see this in the earliest extant references to *dēmokratia* from Aeschylus’ *Suppliants* (ca. 463 BCE), where the Chorus obliquely refers to the “demos’ ruling hand” ( *demou kratousa cheir*, line 604), and “the people which rules the city” ( *to damion to ptolin kratunei*, line 699) ^7^. However parochially circumscribed Athenian citizenship may have been, however much participation within the polity was leveraged through ownership of slaves, and the exclusion of women and non-resident aliens, there is still much to be gained from attending to the ways in which the exercise of democratic power was conceived beyond its institutionalisation or codification.

*Kratos*, as I will define it, expresses a distinctively ‘performative’ mode of power manifesting in moments of prevailing, which encompasses winning an argument, convicting an abuser, turning the tide of battle, reversing a policy decision, conducting a mass boycott, organising a strike, blockading access to disputed territory, or hounding a corrupt leader from office. In all cases, kratic power abides within the *provenness* of authority, as opposed to tethering its legitimacy to institutional mandates or legal precedents. This also makes kratic power far less stable than that which is established through the rule of law or political office. ‘Kratic power’ is never completely detached from institutional contexts, as a theocrat derives authority from divine mandate and doctrinal teachings, and an aristocrat relies upon peer recognition, codes of honour, and aesthetic standards. As for ‘democracy,’ the paradoxical notion of supreme power exercised by unexceptional masses has led theorists like Jacques Rancière and Sheldon Wolin to present the phenomenon as essentially undefinable, a-constitutional, and anarchic ^8^. Although I see the merits of identifying democratic power with the rupturing of established hierarchies, I think it better to avoid making democracy synonymous with ‘revolt,’ as this neglects its specific political form, and compounds the mysteriousness of its persistence within political vocabularies. There have also been illuminating analyses of democratic kratos as a determinedly ‘agonistic’ relation ^9^ or as the cultivation of collective ‘strength.’ ^10^ I will consider each of these readings in turn, using examples drawn from fifth- and fourth-century sources.

 To be clear, my reconstruction of kratos does not intend to offer archaic political forms as solutions for contemporary discontents. As Quentin Skinner has suggested, historical reconstruction serves primarily to “prevent us from becoming too readily bewitched” by prevailing assumptions about the meaning of our norms and political concepts ^11^. Let us proceed, and see whether we can glimpse something approaching an animating spirit for kratos. And what better way to begin than to petition the God himself?

## Divine Kratos

*Prometheus Bound*, the only surviving play of a trilogy attributed to Aeschylus (staged posthumously under the direction of the poet’s son, Euphorion, probably around 430 BCE), opens with the arrival of three gods, bringing the doomed Prometheus in tow. Kratos (‘Supremacy’) is accompanied by his sister Bia (‘Violence’) and Hephaestus, god of the forge, whose unbreakable chains already entrap the rebellious titan. A contrast is immediately established between Prometheus’ stoical silence and the rancorous debate between Hephaestus and Kratos over the justness of Zeus’ punishment:

[66] *Hephaestus*: Ah, Prometheus, I groan for your sufferings!
*Kratos*: Hesitating again, are you? Grieving for the enemies of Zeus? Take care you don’t have cause to pity yourself one of these days!
*H*: Do you see this sight, hard for eyes to look on?
*K*: I see this fellow getting what he deserves. Move down, hoop his legs strongly!
*H*: There, the job is done; the work did not take long.
*K*: Now hammer in the pierced fetters with all your strength; for your work is being assessed by a tough appraiser.
*H*: Your tongue tells the same tale as your appearance.
*K*: You be soft if you want, but don’t make it into a reproach to me that I am implacable and have a harsh temper ^12^.

Note that Kratos, while relishing his role as Zeus’ enforcer, has little or no physical involvement in restraining Prometheus. Kratos exercises power exclusively through the intersubjectively maintained medium of speech ( *commanding* Hephaestus, *indicting* Prometheus, *justifying* Zeus’s right to punish). The manhandling is left to Bia, who silently obeys her brother’s instructions—pinning down Prometheus’ arm, then the other, then his chest and legs—all the while ignoring Hephaestus’ pleas for clemency. Burdened by self-loathing and pity for his divine kinsman, Hephaestus questions Kratos on the need for additional restraints, as this seems cruel and gratuitous. In response, Kratos reminds Hephaestus they are both subject to the exacting standards of a ‘tough appraiser,’ and must do their utmost to ensure Prometheus comes “to accept the tyranny of Zeus” (line 10).

 Danielle S. Allen locates the dispute between the Kratos and Hephaestus within a wider debate about the role of punishment in legitimating authority, particularly as it pertains to spectacular punishments suffered by victims of divine jealousy and wrath ^13^. Where Kratos sees the rightful confirmation of divine order, Hephaestus sees outrageous tyranny. At the same time, there appears no way for Kratos to elicit anything beyond fearful obedience. As the personification of Zeus’ demonstrated superiority, Kratos lacks the foundational or material persistence of territorial possessions, symbolic titles, military assets, and monetary reserves. Whereas Bia actualises her divinity through forceful action, Kratos’ power abides only within the *moment* of prevailing, which is possibly why he seems compelled to taunt Prometheus about how Zeus proved so much “cleverer than he” (line 61). Perhaps it is an appreciation of his own ephemerality that accounts for Kratos’ reluctance to exit the scene with Bia and Hephaestus, and instead remain with the prisoner, basking in the glory of his defeat (lines 79–87).

 Kratos demands to have the last word, irrespective of whether this makes him appear petty or spiteful. There is little else he can do to ensure Prometheus’ imprisonment endures, or that it will be perceived as just. Moreover, Kratos’ loyalty to Zeus persists only for as long as no other divinity proves themselves superior (indeed, it is Prometheus’ premonition that one of Zeus’ own offspring is fated to bring about his overthrow that provides his only source of leverage in appealing his punishment; lines 908–35). Somewhat paradoxically, Kratos’ commitment to ‘perfecting’ Zeus’ triumph over Prometheus’ rebellion—to literally ‘bring it to an end’—cannot assuage the essential ‘imperfective’ aspect of his power, for which he is compelled to habitually re-test and re-assert his supremacy. Despite Hephaestus’ assurances that Prometheus’ arms “have been [permanently] fastened,” Kratos continues issuing imperatives, acutely aware that Prometheus remains “wondrously clever at finding ways out of impossible situations” (line 59–60). Kratos’s supremacy thus entails multiple dependencies, including Zeus’ divine sanction and the supportive essences of his divine siblings Bia (Force), Nike (Victory), and Zelus (Rivalry) ^14^. These divine forces in turn depend upon the public’s recognition of their authority, whether explicitly invoked in the lamentations of the Chorus (who sympathise with Prometheus’ rebellion), or through the spectatorship of Aeschylus’ audience (lines 128–50). For his part, Prometheus remains shackled but hardly seems dominated. In the face of kratic prevailing, he retains the hope for another sudden reversal of fortune. Thus, the domineering character of kratos contains within it the ever-present possibility of turning the tide or retaking control.

 A similar sense of restless instability informs Aristotle’s description of democracy in the *Politics* as a regime-type born of severe inequality and a large underclass, which is also doomed to devolve into untamed score-settling and eventual tyranny:

[1296a.1] [W]here some possess very many things and others nothing, either rule of the people in its extreme form must come into being [ *dēmos éschatos gígnetai*], or unmixed oligarchy, or—as a result of both these excesses—tyranny. For tyranny arises from the most headstrong sort of democracy [ *ek demokratías neanikotátes*] and from oligarchy, but much less often from the middling sorts of regime and those close to them ^15^.

Like the watered-down wine served in communal kraters at elite symposia, Aristotle preferred ‘mixed’ (or more accurately ‘diluted’) constitutions, whose governing institutions are dominated by a well-educated, property-owning middle class. In the absence of such moral-political guidance, there remains only the self-radicalising tendency of the dēmos itself, whose ‘youthful wantonness’ ( *neanikotátes*) ensures recklessness, irrespective of any further manipulation by populist demagogues. In this way, the normative health of a polity cannot be maintained by a self-steering dēmos, because the will of the masses never coalesces around a coherent ideology, and instead remains in a fog of ill-defined impulses, always prone to misdirection.

 We do not need to share Aristotle’s pessimistic appraisal of the political underclass to appreciate the extent to which modern conceptions of democratic legitimacy have largely internalised the necessity of diluting the intemperate will of the dēmos. Rancière describes this condition of modern politics as ‘democracy corrected,’ for which constitutional protections and practices of governance “allow the people to enjoy the visibility of their power through the dispersal and even delegation of their qualities and prerogatives.” ^16^

 For contemporary manifestations of *kratos* we must look instead to mass mobilisations, usually emerging outside established processes, whose bonds of solidarity wax and wane, and must be continually reforged. Such exercises are unlikely to be viewed as ‘constructive’ (in terms of offering new proposals or fashioning new policy instruments) but rather ‘obstructive,’ insofar as they demand accountability and often serve to frustrate governmental initiatives. As we will discuss shortly, the affective demeanour of mass protests can be downright embarrassing. Media coverage of protests are replete with vox pop interviews of strikingly coiffured, red-faced marchers, shouting demands over the din of the crowd. Fearful reports of neo-Nazi infiltrators and ‘black bloc’ agitators abound whenever people take to the streets. These and other discordances are used to discredit collective action as an effective political tool ^17^.

 The amorphousness of ‘mass’ agency makes it hard to identify direct lines of accountability or stabilise contradictory, chaotic impulses around a central aim. But what often motivates these spontaneous, collective actions is a comparable lack of accountability and competence on the part of our leaders and institutions, as the continuing, insufficient responses to climate change, disease pandemics, and police violence can attest. The *kratos* of the ordinary dēmos is primarily a reactive power which can easily turn reactionary. But while some may consider such negativity and obstructionism as a reason to discount the *kratos* of the modern dēmos, to relinquish this threat of wholesale noncompliance would be to abandon any hope of ‘the people’ exerting political control—as even voices within the establishment will concede:

“If you want to pull the party—the major party that is closest to the way you’re thinking—to what you’re thinking, you must— *you must—*show them that you’re capable of not voting for them. If you don’t show them you’re capable of not voting for them, they don’t have to listen to you. I promise you that. I worked within the Democratic Party. I didn’t listen, or have to listen, to anything on the left while I was working in the Democratic Party, because the left had nowhere to go.” ^18^

This brings us to the question of whether the obstructive impulse I identify with kratos can ever be nurtured in such a way as to facilitate a more constructive and stabilised politics. To address this question, we turn to Josiah Ober’s work on the political dynamics of mass and elite actors in ancient Athens, and its attempt to harness a ‘domesticated’ kratos.

## The orator as teacher

Given the cloistered character of academia, it is likely that Josiah Ober is one of the few classicists contemporary political theorists are acquainted with. This is in no small way a result of Ober’s conscientious efforts to bridge traditional disciplinary divides. Nevertheless, while Ober remains an indispensable entry point for understanding contemporary democracies in light of the problems confronting ancient polities, it is equally important to avoid uncritically adopting his conclusions. “The Original Meaning of Democracy” was originally published in *Constellations*, a journal generally devoted to the Frankfurt School tradition of critical social theory, which hopefully augurs well for future cross-pollination between disciplines. Here he offers an alternative reading of democratic power that de-emphasises the domineering character of the masses:

*Demokratia* is not just “the power of the dēmos” in the sense “the superior or monopolistic power of the dēmos relative to other potential power-holders in the state.” Rather it means, more capaciously, “ *the empowered dēmos*”—it is the regime in which the dēmos gains *a collective capacity to effect change in the public realm*. And so it is not just a matter of control of a public realm but the collective strength and ability to act within that realm and, indeed, to reconstitute the public realm through action ^19^.

Ober is in agreement that recovering the ‘original meaning’ of democracy requires focusing our attention on the way agency is structured within the political system *.* A well-functioning democratic regime is built from and reproduces itself through “a socially diverse body of individuals, each capable of choosing freely in his own interests.” ^20^ Yet, despite foregrounding the exercise of power by the masses, Ober grants an outsized role to *elite orators*, whose mediative influence steers the “collective capacity [of the dēmos] to effect change.” Democracy, for Ober, is first and foremost an educative regime, by which political power is effected through *edification* rather than brute imposition:

Athens was a democracy, not just because the ordinary citizen had a vote, *but because he was a participant in maintaining the political culture and a value system that constituted him the political equal of his elite neighbour*. Through publicly performed speech acts, democratic institutions were implicated in *an ongoing process of defining and redefining the truths used in political decision making and of assimilating local knowledges* into an overarching democratic knowledge ^21^.

Here, Ober blunts the jagged edges of contestation so that political aims and identities coalesce around monad-like repositories of democratic ‘knowledge.’ The success of democracy is thereby measured by the legibility of its foundational norms and the willingness of subjects to adopt (and occasionally expand upon) those principles. While such a model grants a clear catalytic role to the orator in shaping opinion and assimilating local knowledges, what is less clear is how the participation of the general citizenry extends beyond attentive spectatorship—or even that there is any evidence to confirm Athenian citizens conceptualised their agency in this way.

 Ober ends the essay with a quote from the fourth century orator, Demosthenes, which supports his contention that the legitimating power of democracy stems from the “relationship between law, action, and the public good”:

[21.225] [T]he laws are powerful [ *ischuroi*] through you and you through the laws.
  You must therefore stand up for them in just the same way as any individual would stand up for himself if attacked; you must take the view that offences against the law are public concerns [ *koina nomizein*].

To provide context, the above passage is taken from one of Demosthenes’ most famous courtroom indictments, in which he accuses his bitter political rival, Meidias, of ‘impious outrage’ ( *hybris*) ^22^. The origins of their dispute date to 348 BCE, when Demosthenes, having been appointed *khoregos* (the public religious official overseeing theatrical productions for the annual Dionysia festival) was allegedly assaulted by Meidias in full view of attending spectators. The attack was the culmination of an extensive campaign of harassment and sabotage by Meidias, who was presumably intent on preventing Demosthenes from receiving a coveted drama prize. In his more extended discussion of the case, Ober describes Demosthenes’ rhetorical strategy as an attempt to frame Meidias’ behaviour as a threat to civic peace ^23^. Knowing his audience was likely composed of citizens with little sympathy for elite rivalries, Demosthenes pointedly underscores that Meidias’ *hubristic* contempt for norms posed a threat to *all* citizens—not least of all those who did not share his own wealth and privilege. However, Ober’s translation cuts Demosthenes off mid-sentence, neglecting this final rhetorical flourish:

[21.225]…you must consider that you share in the wrongs done to the laws, by whomsoever they are found to be committed; and *no excuse*— *neither* public services [ *méte litourgías*], *nor* pity [ *méte éleon*], *nor* personal influence [ *métʼ ándra midéna méte téchnin*], nor forensic skill [ *méte heuristhai*], *nor anything else*—must be devised whereby anyone who has transgressed the laws *shall escape punishment* ^24^.

In Ober’s truncated version, we are left with the impression that Demosthenes intends only to make a high-minded appeal to the rule of law as a public good. But when we view the original passage, the stakes are laid out quite differently, with Demosthenes’ repeated, negative inducements ( *méte*: ‘neither,’ ‘nor’) imploring his audience to block all avenues by which Meidias might *escape conviction*. Alongside whatever pedagogical intent Ober wishes to project, the orator is clearly soliciting the prosecutorial impulse of his listeners. Not only must they condemn the wrongness of Meidias’ actions, they must exercise their juridical power and, to echo the now-familiar refrain, *lock him up*. Contrast this to Ober’s euphemistic framing of democratic agency, in which the orator establishes “the limits of behaviour appropriate to the most powerful individuals in Athenian society [and] the public consequences of allowing those limits to be breached.” ^25^

 Attending to Demosthenes’ language in the above passage also reveals how Ober, in establishing the ‘original meaning’ of *dēmokratia,* quietly substitutes the unwieldy drive of *kratos* with the more harnessable capacity of *strength* ( *ischuroi*), effectively transforming democratic ‘power’ into a virtuous “capacity of a public to make good things happen in the public realm.” ^26^ Although he does not identify any conceptual or etymological link between the two terms, in transmuting the *performative exercise* of power into the *tacit endorsement* of the rule of law, Ober makes the hortatory steering of elite orators an indispensable catalyst for democratic systems. As to whether *kratos* and *ischuroi* are in fact interchangeable, let us consider a different courtroom speech in which Demosthenes actually invokes *kratos*:

[34.19] For it is not the same thing, men of Athens, to give false testimony while face to face with you and to do so before an arbitrator. *With you heavy indignation and severe penalty await those who bear false witness*; but before an arbitrator they give what testimony they please without risk and without shame. […] Lampis, being *so plainly* [ *kata kratos*] *convicted* of bearing false witness and of playing the rogue, admitted that he had made the statement to my partner here, but declared that he was out of his mind when he made it ^27^.

Without getting bogged down in the details of this case (involving an allegation of fraud against two merchants who claimed to have lost a shipment of goods at sea), what is notable here is the way Demosthenes’ rhetorical strategy once again encourages the jurors to be zealous and uncompromising in their verdicts. How else are we to explain the way he invites their indignation, detailing the unscrupulousness of Lampis’ character, and alleging the defendant’s testimony to be so riddled with inconsistencies it could not be presented before a (potentially more sympathetic) private arbitrator, and had to be brought to public trial ^28^. In this instance, kratic power resides not in the jury’s verdict, but in the *probative* incontrovertibility of the evidence against Lampis, and the obviousness of his guilt—in light of which, the jury must once more ensure he does not escape rightful conviction.

 Although Ober leaves open the possibility of an ongoing ‘dialectical give-and-take’ between elite orators and mass audiences, the dynamic he describes suggests a top-down, perfectionist impulse, per which the inculcation of civic morality begets a more normatively attuned citizenry. But this ignores the prosecutorial impulse clearly being elicited by Demosthenes, and his incitement of the jurists’ unceasing vigilance in rooting out the enemies of the polis:

[21.220] [W]henever a solitary victim fails to obtain redress, then each one of you must expect to be the next victim himself, and must not be indifferent to such incidents nor wait for them to come his way, but must rather guard against them as long beforehand as possible.

Ober reduces the agency of the dēmos to a ruminative spectatorship, punctuated occasionally by disgruntled or supportive noises from those seated in the Assembly or *dikasteria* ^29^. Although we cannot hope to divine the feelings and motivations of ancient voters and jurists, we can certainly interrogate Ober’s idealised deliberative model in light of contemporaneous depictions of Athenian juries. What did Athenian citizens and jurists ‘want’ from their power? To pursue this question, let us shift our focus from elite oratory to the bawdy medium of Old Attic Comedy.

## Bdelycleon’s dilemma

Ober understands the social role of Attic comedy as part of a “sophisticated and complex civic ritual” through which the Poet exercises an institutionally circumscribed right to “mock prominent men and to expose the dēmos’ tendency to self-deception.” ^30^ In this, the anarchic incitements of satire remain tempered by the ritualised context of the Dionysia or Lenaea festivals in which they are presented, as well as the Poet’s desire to win the approval of audiences and judges.

 The action of Aristophanes’ fifth century comedy, *Wasps* (first presented in competition at the Lenaea festival of 422 BCE), centres around the troubled home of the retired soldier Philocleon—whose name evokes reverence for the boorish populist Athenian general Cleon. Now in his dotage, Philocleon has become obsessed with volunteering for jury service, as he believes this fulfils his patriotic duty to hunt down corrupt elites and other enemies of the polis. To his son Bdelycleon (whose name suggests physical disgust towards the same Athenian general), the zealotry of Philocleon and his fellow jurists is now a source of considerable embarrassment, and increasingly poses an obstacle to his own upward mobility.

 As the play opens, we learn Bdelycleon has confined his father to home in an attempt to cure him of his courtroom ‘addiction.’ Bdelycleon likens his father’s insatiable prosecutorial fervour to a disease ( *nóson*, line 650), suggesting psychological dependency, for which the continued exercise of power is the problem, rather than any institutional of legal failing. In desperation, Philocleon calls to his fellow *dikasts* for help, and confronts his son with a spirited defence of the jury system. As he regales us with his exploits as a ‘tough appraiser,’ Philocleon enjoys a fleeting resemblance to divine Kratos:

[620] *Philocleon*: So don’t I *wield great authority* [ *megálen arkhèn arkho*], as great as
  Zeus’s? I’m even spoken of in the same way as Zeus
  […] And if I look lightning, the fat cats and the VIPs say a prayer and shit in their pants.
  And you’re very much afraid of me yourself ^31^.

Also worth noting is the way Philocleon invokes *kratos* towards the end of his reverie—not in reference to any accomplishment as a soldier, but rather his own *argumentative* ‘supremacy’ in reducing his learned son (momentarily) to silence:

[635] *Philocleon*: He just thought he’d be ‘picking unwatched vines’ and getting off easy that way. He knew very well that *I’m the boss in this business!* [ *egò taútē krátistós eimi*]

Philocleon’s kratic victory (as always) proves fleeting, and Bdelycleon offers a compelling counterargument (lines 650–710) that his father and the other *dikasts* unwittingly serve the political ambitions of their hero Cleon by zealously persecuting his enemies, all for a paltry three obol salary (line 680). Moreover, he insinuates their ‘jurophilia’ mirrors their earlier unthinking loyalty as soldiers for the Athenian empire (lines 675–80). Then as now, the wasps’ patriotism is taken for granted and poorly compensated, leaving them materially and humiliatingly dependent upon their children. Worst of all, Bdelycleon claims the public trial system is itself ‘rigged’ against meaningful convictions, by virtue of multifarious and lucrative side-dealings between the defenders and prosecutors (line 695). The power Philocleon thinks he wields as a juror is revealed to be at best aspirational (becoming Zeus-like in the eyes of petitioners), and possibly even illusory, since his prosecutorial powers bend to the will of Cleon. Even if these momentary flashes of *kratos* are never wholly false (there is always a perceived loser), this does nothing to ensure subsequent convictions are not a sham. Disputative and prosecutorial victories remain highly unstable, constantly recalibrating to reflect the shifting balance of power. Having punctured his father’s inflated self-regard, Bdelycleon pushes his advantage, entreating Philocleon to abandon public jury-service and accept the substitute of a mock trial, to be staged in the family kitchen, using household objects and pets as witnesses and defendants (lines 800–1000). Bdelycleon’s strategy resembles Ober’s Demosthenes insofar as he uses his elite rhetorical abilities to redirect his father’s prosecutorial fervour and accede to further tutelage in legal reasoning.

 It soon transpires that Bdelycleon’s hopes are severely misplaced, as Philocleon fails to be persuaded by his son’s reasoning against always assuming defendants are corrupt and deserving of punishment. Presented with compelling evidence of the family dog Labes’ ‘innocence’ during the mock trial, Philocleon steadfastly insists upon a guilty verdict. In fact, the only way Bdelycleon is able to ensure his father arrives at the ‘correct’ judgment (in this case, acquitting Labes of allegedly pilfering cheese), is by manipulating evidence, ventriloquising testimony, and finally duping Philocleon into placing his ballot into the ‘wrong’ voting urn (lines 990–4). Having been already ‘shaken to his depths’ by his son’s revelation that courtroom verdicts are regularly overruled by backroom dealings, and that Cleon is “as bad as the rest” of the corrupt Athenian elite, Philocleon is now left utterly despondent by the dog’s playacted acquittal. Once again, Bdelycleon pushes his advantage, goading his father into abandoning jury trials altogether, and eventually convincing him instead to attend an elite symposium. His hope is that Philocleon will develop a taste for sympotic refinements through his careful coaching in social etiquette, conversation, and personal attire (lines 1207–63).

 John Zumbrunnen interprets Bdelycleon’s efforts to rehabilitate his father as illustrative of the unique potential for democratic public life to harmonise competing conceptions of freedom (“rebellious disruption” versus “responsible collective action”) under the gentle rubric of comedic self-recognition ^32^. From this perspective, Aristophanes’ plays are analogous to Demosthenes’ speeches, in that they articulate the duties and benefits of Athenian citizenship in light of emerging conflicts along class and cultural lines. Zumbrunnen views Philocleon and his fellow ‘wasps’ as embodiments of democratic citizenship’s essential paradox: the desire not to be ruled by others engenders a desire to rule others. This defensive/oppressive need cannot be assuaged through institutional processes, but only by cultivating a subtler, comedic disposition to weather the inescapable “contingency, uncertainty, imperfection, and delay” imbuing civic life ^33^.

 I am not convinced, however, that what stirs the anger of the ‘wasps’ is their lack of a ‘firm basis for self-mastery,’ as opposed to the much more straightforward thwarting of kratic prevailing over elites, which results from Bdelycleon’s ‘aristocratic’ efforts to prevent further social embarrassment. In this, I diverge from the conventional reading of Bdelycleon as a relatable protagonist driven by high-minded concerns for democratic civility. As the events play out, it is clear that he is also a self-important social climber driven to transcend his non-aristocratic family background, which requires curbing his father’s prosecutorial activism, and capitalising upon whatever residual social cache Philocleon enjoys as a veteran. Hence, Bdelycleon’s lessons in etiquette come coupled with repeated petitions for his father to exaggerate old war stories, in the hopes this might impress the other guests (line 1187). This plan too fails spectacularly, as his father becomes disgracefully drunk at the elite gathering and, by the final scene, has been declared a madman by the symposiasts, and stands accused of sexual assault (lines 1299–1341; 1484–90). But Philocleon’s ‘rage’ can itself be read as a response to Bdelycleon’s forceful curtailment of his beloved jury service. Denied any meaningful outlet for demonstrating kratic ‘control,’ Philocleon desperately seeks alternative ‘escapes’ (including through the chimney pipe, line 145).

 Even if we impute to Bdelycleon the purest of pedagogical intent, his efforts to ‘perfect’ Philocleon’s moral-political agency bring only anguish and confusion to the old man, hastening his eventual descent into violent animality. It is equally likely that Bdelycleon’s moral pedagogy is an empty conceit, as there is nothing inherently virtuous about ending his father’s ‘patriotic’ jury service, unless we *assume* Philocleon is the guileless henchmen of Cleon, and wrong in his assumption that the very existence of an elite class indicates widespread corruption. Whereas Bdelycleon would seek to avoid embarrassment in pursuit of ‘aristocratic’ status, the democratic power Philocleon wields as a *dikast* is sought without concern for dignity or shame. As such, these two opposing kratic powers are left in mutual incomprehension and contempt. But if Philocleon serves as a cautionary tale of the untamed masses, this should not lead us to ignore the subtler nastiness of Bdelycleon, a caricature of upwardly mobile youth, who over the course of the play reveals himself to be short-tempered dilettante, contemptuous of his social inferiors, and covetous of elite privilege. When Philocleon ‘fails’ to comport with his son’s reformist vision, the mask slips, and the son abandons all decorum, castigating his father as an ‘ignorant oaf’ (line 1183) and an irredeemable ‘pussy grabber’ (χοιρόθλιψ, line 1364). We are left with the unpalatable conclusion that the civic peace sought by moral guardians will never comport with the *kratos* exercises desired by ordinary citizens. And we know that Kratos, once summoned, is incapable of exiting quietly.

 Bdelycleon’s desire to quell his father’s ceaseless prosecutorial impulses echoes contemporary concerns about unrestrained political agency being a threat to ‘true’ democratic sociality ^34^. Indeed, within any nominally democratic society characterised by significant disparities in wealth and status, the desire to ‘hold elites accountable’ inevitably risks demagogic incitement. This is essentially Bdelycleon’s anxiety over his father’s adulation of Cleon (“Which is why I kept you locked up: I didn’t want these blowhards to make a chump of you”; line 720). Again, this assumes that such an implacable, class-based hostility *must* be bad for democracy, and, left unchecked, *must* lead to murderous mob rule, despite the fact that it is Bdelycleon’s own paternalistic interventions that provide the catalyst for his father’s directionless aggression and madness.

 Left alone to their juridical pursuits, was it really inevitable that Philocleon and the wasps would reduce the polis to anarchy? Possibly, but this is only if we ignore Bdelycleon’s own assertion in his debate with Philocleon that verdicts in jury trials are routinely nullified by the secretive agreements between prosecutors and defendants. On the other hand, if reckless juridical *kratos* can be so easily annulled, what danger does it pose to anyone, aside from Bdelycleon’s aristocratic embarrassment? Is the problem with Philocleon’s brash, performative victories that they are *illusory*, or is it that even such ephemeral, epiphenomenal triumphs are capable of inculcating a lasting desire to demonstrate one’s *true* capabilities?

 Returning briefly to Ober’s ameliorative model of deliberative ‘power,’ it is clear he means to dispel the shadow of tyrannical majorities by highlighting the potential epistemic and normative benefits of mass-based political associations. Thus, in his model of fifth- and fourth-century public discourse, the influence exerted by orators like Demosthenes and Cleon was itself reciprocally shaped by the values and expectations of the audience, thereby ensuring the possibility of a virtuous pedagogical circle:

The elite citizen diffused the jurors’ suspicions about the dangerous power that his elite attributes afforded him by humbling himself, by dissimulating his rhetorical skill, by putting his power-producing wealth at the service of the state, by showing that his illustrious ancestors had been highly patriotic, and by affirming that all citizens were of noble birth. For their part, the jurors—and, on a society-wide level, the dēmos as a whole—were persuaded that there was no need to bring their collective political power to bear against the elite. […] *The social stability that resulted from the development of a language of mediation allowed the Athenians to avoid the extreme forms of civil strife that tore apart many Greek states in the late fifth and fourth centuries.* Public rhetoric not only revealed social tension, *it was a primary vehicle for resolving tension* ^35^.

In this, and through the earlier substitution of *ischuroi* for domineering *kratos*, Ober appears to share Bdelycleon’s aim of channelling Philocleon’s ceaseless striving for control to more socially ‘useful’ ends—much like the modern fantasy that imagines redundant coal miners and autoworkers can be retrained as app developers. Yet neither the mock trial, nor Bdelycleon’s attempted inculcation of sympotic refinement offer satisfactory alternatives to the pure restitutive power of kratic prevailing, just as the *dikasteria* was already itself a barely adequate institutional substitute for the wasps’ glory years as soldiers for the empire. Bdelycleon is thus left with a dilemma: every effort to ‘improve’ his father only deepens his resentment and resistance.

 Later in the play Philocleon tells us in an aside that he has been deliberately exaggerating his obtuseness to ‘troll’ his son, and thereby regain some semblance of power through the young man’s consternation (line 1356). Here, it is not a lack of a coherent normative foundation, nor mindless zealotry that constitutes the central problem of Philocleon’s kratic compulsions. Rather, it is democratic society’s inability to simultaneously accommodate the aspirant rule of ‘the best’ *and* the ‘vulgar’ demotic thwarting of elites. Both Philocleon and Bdelycleon are driven by competing kratic impulses: for the father, fulfilling the ‘promise’ of democracy requires proving time and again that it is ‘the people’ are really in charge; for the son, building a ‘better world’ requires the taming such erratic, punitive impulses. Removing this threat of an unembarrassed, demotic *kratos* requires convincing the majority that democracy *can* be achieved through the enlightened mediation of leaders and orators, who are singularly capable of smoothing out the jagged edges dividing democracy from aristocracy. And yet, no containment strategy has proven capable of placating the disquieted dēmos and its unanswered expectation of meaningful political agency. Left unanswered, the *kratos* of the dēmos ruminates along the institutional sidelines, its prosecutorial, obstructive, intemperate force made all the more monstrous by being denied an official space in which to thwart the Promethean designs of their betters. Try as they might to translate *kratos* into the artefactual power of laws, institutions, and adjudicators, democratic leaders seem only to enflame a jealous, kratic refusal to comply. Philocleon represents not only the unembarrassed fury of QAnon and COVID-sceptics, but also the righteous outrage of #MeToo and Black Lives Matter. This leads us to now consider *agonistic* disagreement as the fulcrum for democratic legitimacy.

## Democratic agonism

When I refer to the ‘restrictive’ normativity of contemporary political theory, I mean the studied impatience that arises from expert analyses of mass protest, particularly when discussing the incoherence and naïveté of protester demands. Bdelycleon’s dilemma expresses itself through the perennial philosophical project of impressing onto others what they ought to want from power (howsoever we theorise the varieties of ‘power over’ and ‘power to’). The exertion of epistemic and normative privilege cannot help but be opposed to democratic amorphousness. Turning again briefly to Aristotle, we find in the *Nicomachean Ethics* a troubling thought concerning the power exerted by *kratos* through *enkrateia*, or ‘self-restraint’:

[1146a.10] But a self-restrained man [ὁ σώφρων] must necessarily have strong and evil desires; since if a man’s desires are good, the disposition that prevents him from obeying them will be evil, and so Self-restraint [ἡ ἐγκράτεια] will not always be good; while if his desires are weak and not evil, there is nothing to be proud of in resisting them; nor is it anything remarkable if they are evil and weak ^36^.

Whether the nature of the struggle is against internal compulsions or external obstacles, when harnessing *kratos* it is never enough to simply vanquish the ‘opponent.’ Just as ‘restraint’ is never good in itself, so too any ‘victory’ without opposition remains unremarkable. There must be a *persistent* and *evil* danger for kratic prevailing to hold any merit. Perhaps what makes ‘democracy’ such a compelling political concern is the way this tension has been apparent since the earliest attempts to establish the democratic system:

[1.5] Throughout the world the aristocracy are opposed to democracy, for they are naturally least liable to loss of self-control and injustice and most meticulous in their regard for what is respectable, whereas the many display extreme ignorance, indiscipline and wickedness, for poverty gives them a tendency towards the ignoble, and in some cases lack of money leads to their being uneducated and ignorant ^37^.

What the unknown fifth-century writer (the ‘Old Oligarch’) fails to acknowledge is that even an undisciplined *kratos* wielded by ignorant masses still constitutes true *enkrateia*, insofar as they are demonstrably able to ‘restrain’ the actions of contemptible elites.

 For the contemporary Bdelycleon aristocrat, the imperative is to tame or otherwise isolate the braying, ignorant demands of the masses for the sake of societal stability. Hortatory steering serves both to edify and corral non-experts away from direct access to power. For the contemporary Philocleon democrat, the aperture for freely exercising political control remains maddeningly out of reach. The masses must therefore fanatically defend their diminished agency (e.g., as consumers), while also desperately seeking new ways to thwart the designs of the professional managerial class. Thus, the demonstrative moment of *kratos* inevitably gives way to the problem of ensuring this power can be perpetuated and renewed. These tensions are not reducible to a balancing of political ‘spontaneity’ versus ‘stability.’ Rather, as we have seen, what perpetuates kratic conflict is the performative need *to be seen to prevail* over obstacles and opponents. Proponents of ‘agonistic democracy’ have argued along similar lines ^38^. From this perspective, ‘democracy’ is an ongoing struggle to define the ‘political’ character of the dēmos:

[T]he aim of democratic politics is to transform *antagonism* into *agonism* This requires providing channels through which collective passions will be given ways to express themselves over issues which, while allowing enough possibility for identification, will not construct the opponent as an enemy but as an adversary. An important difference with the model of ‘deliberative democracy’ is that for ‘agonistic pluralism,’ the prime task of democratic politics is not to eliminate passions from the sphere of the public, in order to render a rational consensus possible, but to mobilise those passions towards democratic designs ^39^.

Although I would suggest that *kratos* arises from the same, essentially political ‘dimension of antagonism inherent in human relations’ underlying ‘ordinary politics,’ ^40^ what distinguishes kratic power is its resistance to ‘domesticating’ this antagonism into agonism. If Ober’s dialectic of ‘mass and elite’ mirrors Bdelycleon’s efforts to cultivate sympotic manners in his father, Mouffe’s ‘conflictual consensus’ resembles the playacted trial of the family dog, Labes. In both cases, unseemly antagonisms are sublimated by provisioning of space for ‘legitimate’ ideational contests and cultivating ‘respect’ between partisans ^41^. In my own reading of *kratos*, struggle is not an end in itself, but merely one possible means to ensure one’s *prevailing*. As we recall from the examples of Kratos versus Prometheus, and Bdelycleon versus Philocleon, kratic prevailing entails *nullifying* an opponent’s *evil* objectives, even if this victory comes at the expense of realising a preferred set of objectives. Thwarting Prometheus’ unsanctioned gift of divine knowledge to humanity; preventing Philocleon from hounding a corrupt politician from office or making them suffer *atimia*—these negations are not carried out with a view to improving argumentation or institutional procedures. What matters for *kratos* is *winning*, whatever the cost.

 Another reason for resisting a linguistified rendering of *kratos*—even within the ceaselessly oratorical culture of ancient Athens—is that it misconstrues the decidedly non-deliberative nature of mass agency in general, and Athenian decisional processes in particular (which remained distinct from the counsel of the orators). As far as exercises of fourth- and fifth-century democratic kratos are concerned, we can be relatively certain that deliberation in the *ekklasia* and popular courts was *internally* directed; that is, voters and jurors ‘made up their minds’ about an issue (e.g. to vote against a proposed law, or in favour of imposing *atimia* or ostracism) for which they held final decisional authority, yet were not expected to arrive at their judgments through an exchange of reasons ^42^. Contrary to the agonistic conception of being *seen* to ‘take a stand,’ the institutionalised processes by which ancient kratic power was wielded (so that the dēmos held the polity firmly ‘in its grip’ ^43^) tended to preserve the anonymity of individuals, both in decisions reached through a ‘show of hands’ ( *cheirotonia*) and through the casting of potsherds ( *psephophoria*) ^44^.

 There is also a lingering Bdelycleon perfectionism in agonistic accounts whereby ideological ‘chains of equivalence’ are forged in order to harmonise discordant demands and steer collectives toward unifying objectives. Regardless of how loosely federated hegemonies are expected to be, the motivating impulse of democratic agonism remains that of defusing the danger posed by untutored masses, and preventing their being entranced by hegemonic visions that run counter to their ‘real’ interests ^45^. Ideology critique has its uses, but we should not just assume the unrefined, unstable, negating power of kratos (which underlies the collectivised obstructionism of the strike or blockade) renders it politically deficient or secondary to true partisan struggle. Nor should we presume the only legitimate form of political agency is that which remains ‘answerable’ for its supposed beliefs. The Aristotelian charge that a leaderless dēmos inevitably lapses into tyranny ignores the fact that the ‘restraint’ of meta-ethical clarity is not a good in itself—and indeed renders a dēmos less capable of stopping imminent calamity—for fear their actions might prove insufficiently universalisable. ‘Clarifying’ ascriptions of ideology or partisanship are also capable of exacerbating confusion about what the collective desires or represents—recall Philocleon’s destructive bewilderment upon having his worldview ‘corrected’ by Bdelycleon’s critical unmasking of the *dikasteria* as a demagogic tool. Which is not to say that collective kratic actions are inherently superior to partisan struggle or representative democracy, only that such efforts at political dressage must also prove capable of ‘winning’ something more than argumentative or moral victories. In my concluding thoughts for this paper, I will consider what relevance, if any, my reconstruction of ancient kratos may have for discussions of contemporary political discontents.

## Kratos unbound?

In this paper I have sought to describe a distinctive mode of power I identify with *kratos*, which consists in efforts to paralyse, perplex, and prevail over perceived obstacles or opponents. I have also sketched the peculiar dynamic by which enlightened thinkers and policymakers have sought to ameliorate the violent, destabilising tendencies of kratic power, as wielded by a dēmos *.* Whether through forceful curtailment, incentivised redirection, or moral didacticism, attempted domestications are invariably tied to a concern that the kratic demand to *exercise* control cannot be extended to the intemperate, unaccountable masses. A leader must step forward, a group must cohere around a set of negotiable demands. However, I also contend that this fails to learn the lessons of Bdelycleon, and fails to see how pedagogical and hortatory influence can be as corrupting and dangerous as any threat of mob violence.

 In contrast to the aristocratic, ‘perfectionist’ tendency, the restlessness of demotic *kratos* is more conducive to the unresolved, *imperfective* grammatical aspect, per which an action remains unfinished, habitual, and indeterminate in its duration, frequency, and recurrence ^46^. It is only for *as long as* Indigenous land defenders manage to block pipeline workers from entering unceded Wet’suwet’en territory; or anticapitalist protestors hold off police forces from clearing improvised encampments; or housing activists prevent landlords from entering eviction court, that demotic *kratos* can be said to prevail. *Kratos* suffuses the resolute demands of Black Lives Matter that promises of reform much be met with *proof*, in the actual firing and conviction of murderous officers, or the defunding of departments that have too long tolerated their abuse. And *kratos* reappears in the moment #MeToo morphs from a trending topic on social media to unprecedented legal actions against alleged sexual predators.

 To theorise democratic power ‘in the imperfect’ requires surrendering the interpretive authority by which leaders, representatives, assorted commentators, and theorists try to ‘perfect’ political actions by defining ‘once and for all’ their extensiveness and implications. Typically, demands that issue from the position of the powerless are considered to be tainted by the violence of desperation, or the untutored simplicity by which political problems are cast into crude oppositions (“with us or against us,” “part of the problem or part of the solution”). But there is good reason for this entrenchment, as it is all too easy for isolated actors to be either silenced or co-opted by the very powers they castigate.

 The shared antipathy of a dēmos against the *áristos* can also encompass any number of overlapping and incompatible plaints—from antisemitic or racist conspiracy mongering, to vulgar class reductionism; from anti-corruption zealotry, to pure anarchism. But it does not follow from these incoherencies that kratic obstructionism is inherently inferior to party-disciplined, piecemeal reformism. The kratic dēmos grows restless under Bdelycleon domestication and is increasingly contemptuous of self-appointed Promethean visionaries. Though it may be futile to seek an outlet for unmediated democratic power within existing institutions (and undesirable to romanticise ‘abject’ political agency), I believe there is much to gain from reminding ourselves that such proscriptions have been chosen, if not by us personally. Kratos resides in an imperfective world, always provisional, always seeking to challenge, always intent on resuming unfinished business.

 When in 428/27 BCE the Athenian Assembly voted, initially in favour, and then against, the punitive enslavement and execution of the entire population of Mytilene (the largest of the *poleis* of Lesbos which had revolted against Athenian imperial rule), the aforementioned populist blowhard Cleon castigated the citizenry for its failure to demonstrate tractability in following what he knew to be ‘the good’ of the polis.

[3.37.3] But quite the most alarming thing is, if nothing we have resolved upon shall be settled once for all, and if we shall refuse to recognise that a state which has inferior laws that are inviolable is *stronger* [κρείσσων] than one whose laws are good but without authority; that ignorance combined with self-restraint is more serviceable than cleverness combined with recklessness; and that simpler people for the most part make better citizens than the more shrewd ^47^.

Even for a populist like Cleon, the intemperance and changeability of the dēmos gives reason to despair. Such fickleness and pseudo-sophistication is better off being entirely supplanted by loyal, incurious complacency. We will never know to what extent the Assembly’s decision to reverse its original order to slaughter the Mytileans was influenced by the counsel of Cleon’s opponent, Diodotus; whether it was driven by genuine regret, or was simply a random effect of its changing daily makeup—what matters is that ‘the people,’ however ill-advisedly, had ‘the last word’ on the matter. In contrast, we might consider the utter lack of *kratos* exerted by the unprecedented millions who marched against the 2003 US-led invasion of Iraq ^48^. For better or worse, neither the administration of justice, nor the execution of military misadventures are beholden to popular consent. Representative democracy, particularly in the US, is a gerontocracy. seemingly impervious to change even in the face of a disease pandemic and extinction-level environmental catastrophe ^49^. Whatever the merits of representative translations of unruly kratic demands, there is little indication that popular initiatives have any institutional purchase (hence the noticeable lack of any mention of debt relief or police reform during the 2020 Democratic National Convention). The responsiveness of representative institutions is being tested and found wanting. I do not think *kratos* is neatly reducible to left or right populism, nor do I perceive any clear institutional channels for satisfying kratic intemperance (i.e., through mini-publics or a relinquishing of state control to grassroots networks). My abiding critical concern is the aristocratic dismissal and denigration of popular control within a *nominally* democratic system—which I believe is only certain to produce more Philocleon monstrosities. Left unheard and unanswered, kratic resentment against elites putrefies into Pizzagate and ‘Q’-research, until such time that the diseased prosecutorial fervour bursts back into the mainstream ^50^. Should *kratos* ever be retaken by the dēmos, it will likely prove wanton and unreliable. Yet it is also true that nothing less than a mass mobilisation, forcing the curtailment of extractive industries and the removal of corrupt sinecures, can ensure the survival of our species. We find ourselves at the mercy of forgotten gods.

## Data availability

No data are associated with this article.

## Footnotes

^1^ Urbinati, Nadia. 2019. *Me the People: How Populism Transforms Democracy*. Cambridge and London: Harvard University Press; Revelli, Marco. 2019. *The New Populism: Democracy Stares into the Abyss*, tr. by David Broder. London and New York: Verso; Müller, Jan-Werner. 2016. *What is Populism?* University of Philadelphia Press.

^2^ Transcript of June 13, 2020 press conference with New York Governor Andrew Cuomo: https://news.yahoo.com/cuomo-don-t-protest-won-163708594.html

^3^ Lindeman, Tracey. 2020. “Justin Trudeau tells Canada protesters: ‘The barricades need to come down’” *The Guardian* (21.02.2020): https://www.theguardian.com/world/2020/feb/21/justin-trudeau-canada-rail-blockades

^4^ Kipnis, Laura. 2017. *Unwanted Advances: Sexual Paranoia Comes to Campus*. New York: HarperCollins (17).

^5^ Regarding extant sources for this etymology, see: Raaflaub, Kurt. 2007. “The Breakthrough of *Dēmokratia* in Mid-Fifth-Century Athens,” In *Origins of Democracy in Ancient Greece*. Kurt Raaflaub, Josiah Ober, Robert Wallace (eds.) Berkeley, Los Angeles, London: University of California Press (pp. 107–113).

^6^ Pew Research Center. 2019. “European Public Opinion Three Decades After the Fall of Communism”: https://www.pewresearch.org/global/2019/10/15/european-public-opinion-three-decades-after-the-fall-of-communism/

^7^ Aeschylus, *Suppliants*, in *Aeschylus - Volume I*, trans. Alan H. Sommerstein. Loeb Classical Library 145, Cambridge, MA: Harvard University Press, 2008; On the translation of *demou kratousa cheir* as “dēmos’ ruling hand,” see: Cartledge, Paul. 2007. “Democracy, Origins of: Contribution to a Debate,” in *Origins of Democracy in Ancient Greece* (pp. 155–69).

^8^ Rancière, Jacques. 1990. *On the Shores of Politics*, trans. Liz Heron. London and New York: Verso; Wolin, Sheldon S. 2016. “Norm and Form: The Constitutionalising of Democracy,” in *Fugitive Democracy and Other Essays*. Princeton and Oxford: Princeton University Press.

^9^ Mouffe, Chantal. 2000. *The Democratic Paradox*. London and New York: Verso.

^10^ Ober, Josiah. 2008. “The Original Meaning of ‘Democracy.” *Constellations* 11, No. 1: 3–9.

^11^ Skinner, Quentin. 1998. *Liberty Before Liberalism*. Cambridge University Press (p. 116).

^12^ Aeschylus, *Prometheus Bound*, in *Aeschylus - Volume I*, trans. Alan H. Sommerstein. Loeb Classical Library 145, Cambridge, MA: Harvard University Press, 2008.

^13^ Allen, Danielle S. 2000. *The World of Prometheus: The Politics of Punishing in Democratic Athens*. Princeton University Press.

^14^ Hesiod. 2018. *Theogony*. Translated by Glen W. Most. Loeb Classical Library 57, Harvard University Press (§383).

^15^ Aristotle. 1944. *Politics*. Translated by H. Rackham. Loeb Classical Library 264. Harvard University Press.

^16^ Rancière, Jacques. 1995. *On the Shores of Politics*. Translated by Liz Heron. London and New York: Verso: 95.

^17^ On the complex (and mostly counterproductive) relationship between activists and media outlets, see: Sobieraj, S. 2011. *Soundbitten: The Perils of Media-centered Political Activism*. New York: New York University Press; and Gitlin, T. 2003. *The Whole World is Watching: Mass Media in the Making and Unmaking of the New Left*. Berkeley: University of California Press.

^18^ Lawrence O’Donnell. 2006. Transcript of interview with MSNBC host (former Democratic Chief of Staff for Senate Committee on Finance, 1993–95), in *An Unreasonable Man*. PBS/IFC Films (2006); transcript: https://www.counterpunch.org/2007/12/26/two-unreasonable-men/.

^19^ Ober 2008: 7, emphasis added.

^20^ Ober, ibid.

^21^ Ober, Josiah. 1994. “How to Criticise Democracy in Late Fifth- and Fourth-Century Athens,” in Euben, Wallach, Ober (eds.) *Athenian Political Thought and the Reconstruction of American Democracy*. Ithaca and London: Cornell University Press): 163–4; emphasis mine.

^22^ For background on this case: Harris, Edward M. *Demosthenes: Speeches 20–22*. Austin: University of Texas Press, 2008 (75–87).

^23^ Ober, Josiah. 1996. “Power and Oratory in Democratic Athens: Demosthenes 21, *Against Meidias*.” In *The Athenian Revolution: Essays on Ancient Greek Democracy and Political Theory*. New Jersey: Princeton University Press: 86–106 (96).

^24^ Demosthenes. 1935. *Orations Volume III (21–26)*. Translated by J.H. Vince. Loeb Classical Library 299, Cambridge, MA: Harvard University Press; emphasis added.

^25^ Ober 1996: 93.

^26^ Ober 2008: 8.

^27^ Demosthenes. 1936. “Against Phormion” (ca. 327/6 BCE), in *Orations Volume IV (27–40)*. Translated by A.T. Murray. Loeb Classical Library 318, Cambridge, MA: Harvard University Press; emphasis added.

^28^ For background on this case: MacDowell, Douglas M. 2004. *Demosthenes, Speeches 27–38*. Austin: University of Texas Press (110–15).

^29^ For a discussion of the frequency and occasionally strategic use of heckling, see: Hansen, Mogens Herman. 1987. *The Athenian Assembly in the Age of Demosthenes*. Oxford: Basil Blackwell (pp. 69–72).

^30^ Ober, Josiah. 1998. *Political Dissent in Democratic Athens: Intellectual Critics of Popular Rule*. Princeton: Princeton University Press: 122–6.

^31^ Aristophanes. 1998. *Wasps* [c.422 BCE]. In *Aristophanes - Volume II*. Translated by Jeffrey Henderson. Loeb Classical Library 488. Cambridge, MA: Harvard University Press; emphasis added.

^32^ Zumbrunnen, John. 2012. *Aristophanic Comedy and the Challenge of Democratic Citizenship*. Rochester: University of Rochester Press.

^33^ Zumbrunnen 2012: 131.

^34^ See, for example: Urbinati, Nadia. 2019. *Me the People: How Populism Transforms Democracy*. Cambridge, MA and London: Harvard University Press.

^35^ Ober 1989: 307–8; emphasis added.

^36^ Aristotle. 1926. *Nicomachean Ethics*, tr. H. Rackham. Loeb Classical Library, Harvard University Press.

^37^ Xenophon? 1975. *The Constitution of the Athenians*, trans. JM Moore. University of California Press (p. 38).

^38^ See: Vardoulakis, Dimitris. 2017. “Stasis: Notes Toward Agonist Democracy” *Theory & Event* 20:3 (July 2017): pp. 699–725; William E. Connolly. 2005. *Pluralism*. Duke University Press.

^39^ Mouffe 2000 (p. 103).

^40^ Mouffe 2000 (p. 101).

^41^ Connolly 2005 (pp. 122–4).

^42^ On the etymological trajectory of ‘deliberation’ [ *bouleuomai*] in the fourth- and fifth- centuries, which saw a conventional shift towards the ‘middle voice’ in describing the activity of the listening audience seated in the Assembly (as opposed to the ‘advisement’ [ *symbouleuo*] the orators), see: Cammack, Daniela. 2020. “Deliberation and Discussion in Classical Athens,” *The Journal of Political Philosophy*: https://doi.org/10.1111/jopp.12215

^43^ ‘Kratos’ is used in this manner by the Aristotelian author of the fourth-century *Athênaiôn Politeia*, where, following its defeat at the end of the Peloponnesian War in 404 BCE, Athens fell “firmly in the grip” ( *tên polin engkratesteron eschon*) of the pro-Spartan ‘Thirty’ through its campaign of mass arrests and executions. [?Aristotle. 1935. *Constitution of the Athenians*, tr. H. Rackham. Loeb Classical Library 285. Harvard University Press. (§35)].

^44^ On the complexity of Athenian voting technology, and the way in which the anonymity of jurors was facilitated by innovations like the *kleroterion*, see: Hansen, Mogens Herman. 1999. *The Athenian Democracy in the Age of Demosthenes*. Norman: The University of Oklahoma Press (pp. 200–3).

^45^ Mouffe, Chantal. 2018. *For a Left Populism*. London and New York: Verso (pp. 20–4).

^46^ For a more elaborative discussion of ‘moral imperfectionism,’ see: Leela Gandhi. “Utonal Life,” in *Cosmopolitanisms* (eds. Bruce Robbins and Paulo Lemos Horta) New York: New York University Press.

^47^ Thucydides. 1930 *History of the Peloponnesian War*, tr. Charles Forster Smith. Loeb Classical Library (p. 59); For illuminating analysis of the competing rhetorical strategies of Cleon and Diodotus, see Yunis, Harvey. 1996. *Taming Democracy: Models of Political Rhetoric in Classical Athens.* Cornell University Press (pp. 87–101).

^48^ Fishwick, Carmen (and *Guardian* readers). 2016. “‘We were ignored’: Anti-war protestors remember Iraq war marches,” *The Guardian* (08.07.2016): https://www.theguardian.com/uk-news/2016/jul/08/we-were-ignored-anti-war-protestors-remember-the-iraq-war-marches

^49^ According to a Congressional Research Service report following the 2018 midterms, the average age of Members of the House is 58, while in the Senate the average age is 62. Nancy Pelosi (speaker of the House) and Mitch McConnell (majority leader in the Senate) are 80 and 78 years old, respectively. President Donald Trump, who turned 74 in June 2020, was already the oldest US president upon being elected to his first term, and is now fighting for re-election against Senator Joe Biden, who will be 78 this November ( https://www.senate.gov/CRSpubs/b8f6293e-c235-40fd-b895-6474d0f8e809.pdf)

^50^ E.g. the currently 24 Congressional candidates (22 Republicans, 2 Independents) who have been outspoken in their endorsement of a conspiracy theory (disseminated anonymously online through message board sites and Facebook groups) that President Donald Trump is working secretly with John F. Kennedy Jr. to purge the United States government of an elite cabal of Satanists and paedophiles overseeing the ‘deep state.’ Alex Kaplan, a reporter for the non-profit media watchdog Media Matters for America, has been keeping a running tally of current and former congressional candidates that support the self-identified intelligence operative known as ‘Q’: https://www.mediamatters.org/qanon-conspiracy-theory/here-are-qanon-supporters-running-congress-2020
