# What’s Wrong with Democracy? The Peculiar Power of *Kratos*

**DOI:** 10.12688/openreseurope.13726.2

**Published:** 2022-11-28

**Authors:** John McGuire

**Affiliations:** 1 School of Philosophy, University College Dublin, Belfield, Dublin, Dublin 4, Ireland

**Keywords:** Ancient and Modern Democracy; Democratic power and discontent; Populism; Demosthenes; Aristophanes; Aeschylus; Josiah Ober

## Abstract

In this paper, I reconstruct the notion of *kratos* as a unique and distinguishable mode of political power. My claim will be that *kratos* encapsulates a specific problem for democracy, in both ancient and modern contexts. Using examples from 5th- and 4th- century Attic tragedy, Old Comedy, and forensic oratory, I show how *kratos* was used in Athenian cultural and political discourse to convey the *irrefutability* of a claim, the recognition of *prevailing* over another, and the sense of *having the last word*, all of which makes kratic power dependent upon its continued demonstrability. Although the agency of the dēmos is now largely mediated through representative mechanisms, the peculiarly kratic drive towards ‘winning,’ with all its inherent instability and vacuity, remains indispensable for democratic legitimacy. In focusing critical attention on *kratos* and the pathology of winning, I hope to shift discussion from what divides ‘elites’ from the ‘masses,’ to a problem shared in common.

The aim of this paper is to reconstruct one of the basic intuitions underpinning ancient Athenian democracy, specifically the motive power expressed by *kratos*. My claim is that *kratos* encapsulates a specific problem for democracy in both ancient and modern contexts. Whereas political philosophy has tended to exhaustively analyse the dēmos (identifying, predicting, translating, and educating the actions of the electorate), comparatively little attention has been paid to what this collective agent seeks to *do* with its power. As we will see, *kratos* is both indispensable to democracy and essentially vacuous: it is the problematic power granted by ‘winning.’

## (1) Defining the problem

For better or worse, Attic coinages continue to serve as anchoring terms in Western political discourse. Not only the general category of our politics (τὰ πολιτικά), but many of its most contentious labels are phonetic approximations of this same Ionian dialect: αὐτόνομος (autonomous), βάρβαρος (barbarous), δημοκρατία (democracy), δεσπότης (despot), κυνικός (cynical), μανία (mania), ὀλιγαρχία (oligarchy), συκοφάντης (sycophant), τύραννος (tyrant). And while opinion polls continue to suggest widespread popular support for norms linked to democracy (including gender non-discrimination and equality before the law), most remain at a loss to explain how their own agency feeds into this system. ^1^ Indeed, a growing cohort of voters in the US appear committed to the belief that voting is hopelessly ‘rigged,’ and support candidates who openly express the desire to reverse the results of the 2020 Presidential election. ^2^ As outlets for democratic expression are narrowcasted to voting and occasionally canvassing for one’s preferred candidate, democratic institutions have come to resemble esoteric grimoires, who workings are discernible only to a select group of technocrats, pundits, and theorists. In turn, we have seen elaborate conspiracy theories taking the place once occupied by foundational nationalist myths. ^3^

 By contrast, when we consider the workings of ancient democracy (at least as experienced by the privileged class of men who could claim political membership) we imagine a much more direct and appreciable experience of power. Part of this accessibility is reflected in language, where novel political coinages of the Attic period appear decomposed into parts and synecdochally grasped. At least this seems to be the case with the earliest extant reference to *dēmokratia*, in Aeschylus’ *Suppliants* (ca. 463 BCE), where the Chorus obliquely refers to the “dēmos’ ruling hand” ( *demou kratousa cheir*, line 604), and “the people which rules the city” ( *to damion to ptolin kratunei*, line 699). ^4^ However parochially circumscribed Athenian citizenship may have been, however much participation within the polity was leveraged through ownership of property and slaves, or the exclusion of women and non-resident aliens, there remained an expectation of unmediated control by ordinary citizens.

*Kratos*, as I will define it, evinces a distinctively performative power, manifested in moments of prevailing: winning an argument, convicting an abuser, reversing the tide of battle, overturning a policy decision, conducting a general strike, blockading access to disputed territory, or hounding corrupt leaders from office. ^5^ In all such cases, *kratos* abides within the momentary provenness of superior power, independent of any institutional mandate or legal precedent. ^6^ This suggests kratic power is far less stable than the kinds of authorisation secured through the rule of law or political office. Still, *kratos* depends upon a supportive context to have any meaning: a theocrat bases strict hierarchical control upon doctrinal teaching and ritual; and the aristocrat relies upon peer recognition, codes of honour, and aesthetic standards. *Kratos* is equally at home within aristocracy, democracy, or theocracy; its only fidelity is to ‘triumph,’ whatever the cause. Paradoxically *kratos* is both undeniable and meaningless; it embodies demonstrable proof yet expresses no legitimacy beyond the happenstance of the ‘win.’

 I am focusing upon this component of our inherited political vocabulary because I believe *kratos* illuminates an ‘unsolved’ problem that has carried over into modern democratic life. In this, I take my lead from Hannah Arendt, who does not look to Athens to recuperate some heroic past but to understand how conceptual transformations, omissions, and exclusions have shaped the exigencies of the present. ^7^ In a similar vein, Quentin Skinner sees historical reconstruction as helping “prevent us from becoming too readily bewitched” by prevailing assumptions about our guiding norms and political concepts. ^8^ Although I will offer evidence of my claims using representative examples from ancient sources, I am in no way claiming to present a thorough semantic history. ^9^ I appreciate that engaging with the corpus in this limited way may seem too truncated to garner true historical insight. Nevertheless, I believe recovering some semblance of how *kratos* was earlier understood is a necessary starting point for rethinking the ‘problem’ of democracy today.

Contemporary critiques of democratic populism typically fall into the categories outlined by the 5th-century BCE pseudonymous author of the *Constitution of the Athenians*:

[1.5] [E]verywhere on earth the best element is opposed to democracy. For among the best people there is minimal wantonness and injustice but a maximum of scrupulous care for what is good, whereas among the people there is a maximum of ignorance [ *amathia*], disorder [ *ataxia*], and wickedness [ *ponēria*]; for poverty draws them rather to disgraceful actions, and because of a lack of money some men are uneducated and ignorant.

We do not need to share Pseudo-Xenophon’s pessimistic appraisal of the political underclass to appreciate the extent to which modern conceptions of democratic legitimacy have internalised the need to dilute the intemperance of the dēmos. Jacques Rancière describes this condition of modern politics as ‘democracy corrected,’ for which constitutional protections and practices of governance “allow the people to enjoy the visibility of their power through the dispersal and even delegation of their qualities and prerogatives.” ^10^ Complaints about *amathia* re-emerge in contemporary writers like Jason Brennan, who heaps scorn upon the guilelessness of ‘Hobbits’ and ‘Hooligans’ (in Brennan’s clumsy typology), for which he proposes greater deference to the resolute expertise of ‘Vulcans.’ ^11^ Those unpersuaded by the call for epistocracy see equivalent dangers arising from the structural *ataxia* of the electoral system, and see viciously competitive partisanship as the real instigator of civil unrest. From this, proponents of sortition and pluralistic coalition-building aim improve the quality of democratic decision-making by removing the toxicity of electioneering altogether. ^12^ Finally, concerning *ponēria*, we regularly hear of the essential ‘wickedness’ of populist deifications of ‘The People’ and the conflation of normative legitimacy with merely ‘factual’ success. ^13^ In all such cases however, it is the dēmos that targeted for improvement. My aim is to shift critical attention to *kratos* because, as I will argue, even the most rational, responsive, pluralist version of a dēmos still contends with the perverse incentives and compulsions of empowerment-through-prevailing. I believe a reconstituted conception of *kratos* best encapsulates the dilemma contemporary democracies face: on the one hand, the vast majority of citizens lack any experience of unmediated political control. On the other hand, those who hold power remain preoccupied with safeguarding their ‘electability.’ Thus, even when *kratos* has all but completely been siphoned from the dēmos, the compulsion to ‘win’ and ‘prevail’ overwhelms all other concerns.

 The problem posed by *kratos*, is therefore different to the problem posed by populism, insofar as the preternatural drive towards winning overrules identarian concerns with purity or authenticity. ^14^ Whereas *kratos* abides only within the moment of its supremacy, populism can easily be nourished in the soil of failure and *ressentiment* (which is not to reduce populism to psychological regression, but to emphasise its resilience through changing political fortunes). The reign of *kratos* extends no further than the heady immediacy of being ‘on a roll,’ distinct from any lasting or residual association with a team or party ^15^ As we will see, *kratos* must always be unhesitatingly assured of its own power; it cannot be mediated, sublimated, or satisfied by substitutes. And it is this experiential, tactile quality that contrasts with the ‘us versus them’ animus of populist demagogues. Perhaps too, it is the indiscriminate orientation towards ‘victory’ that makes *kratos* suited to the agency of an undifferentiated dēmos, despite there being so little room within modern representational democracies to manifest the spirit of vanquishment.

 The paradoxical notion of a supreme power exercised by unexceptional masses has led theorists like Jacques Rancière and Sheldon Wolin to suggest democracy is essentially undefinable, a-constitutional, and anarchic. ^16^ While I see the merits of identifying democratic power with the ‘rupturing’ of established hierarchies, I want to avoid making democracy synonymous with ‘revolt,’ as this does not help us see how *kratos* repeatedly implicates both powerbrokers and the powerless. The vision of democracy held by Wolin and Rancière is attuned to different kind of ‘power’ that intervenes in such a manner to have lasting consequences (either a more inclusive form of participation, or a dissolution of entrenched hierarchies). The *paradoxical* power I am identifying with *kratos* functions differently, not only by being more frequent and militant than ‘fugitive’ democracy, but also by being essentially inconclusive and ineffectual. Political victories—particularly electoral victories—are not ‘legacies,’ each ‘turning of the tide’ provides a narrative twist without a conclusion. We should interrogate the presumption that successive re-elections offer any substantive lesson or obvious principle. ‘Winning’ is a wholly distinct phenomenon from *maintaining* power, especially to those dwelling on the political sidelines, for whom it remains a mystery what, aside from winning, is being accomplished. As we will see, the problem *kratos* creates for democracy does not stem from the supposed ungovernability of the masses. Equally hubristic is the elitist impulse to contain, educate, or steer the unruly masses. The problem with *kratos* lies in the *incessancy* of its validating power. Let us proceed then, and see whether we can gain a clearer grasp of this animating spirit. And what better way to begin than to petition the God himself?

## (2) Divine Kratos

*Prometheus Bound*, the only surviving play from a trilogy attributed to Aeschylus (staged posthumously under the direction of the poet’s son, Euphorion, probably around 430 BCE), opens with the arrival of three gods, bringing the condemned Prometheus in tow. Kratos (‘Supremacy’) is accompanied by his sister Bia (‘Violence’) and Hephaestus, the god of the forge, whose unbreakable chains already constrain the rebellious titan. A contrast is immediately established between Prometheus’ stoical silence and the rancorous debate between Hephaestus and Kratos over the justness of Zeus’ punishment:

[66-80] *Hephaestus*: Ah, Prometheus, I groan for your sufferings!
*Kratos*: Hesitating again, are you? Grieving for the enemies of Zeus? Take care you don’t have cause to pity yourself one of these days!
*H*: Do you see this sight, hard for eyes to look on?
*K*: I see this fellow getting what he deserves. Move down, hoop his legs strongly!
*H*: There, the job is done; the work did not take long.
*K*: Now hammer in the pierced fetters with all your strength; for your work is being assessed by a tough appraiser.
*H*: Your tongue tells the same tale as your appearance.
*K*: You be soft if you want, but don’t make it into a reproach to me that I am implacable and have a harsh temper. ^17^

Note that Kratos, as Zeus’ enforcer, appears to have little or no physical role in restraining Prometheus. Although there are no explicit stage directions, the dialogue allows us to surmise that Kratos is exercising his authority primarily through the medium of speech: commanding Hephaestus, indicting Prometheus, rationalising Zeus’ right to punish. For her part, Bia stays silent in obeying her brother’s command to pin down Prometheus’ arms, chests, and legs—manifesting a purely *physical* power. Meanwhile, burdened by self-loathing and pity for his divine kinsman, Hephaestus petitions Kratos against the need for additional restraints, which he believes serve no purpose other than gratuitous cruelty. Kratos reminds Hephaestus they are both subject to the exacting standards of a ‘tough appraiser,’ reinforcing his assertion at the opening that Prometheus must be made to “accept the tyranny of Zeus” (line 10).

 Danielle S. Allen locates this dispute between the Kratos and Hephaestus within a wider debate about the role of punishment in legitimating authority, particularly the spectacular punishments suffered by victims of divine jealousy and wrath. ^18^ Where Kratos sees the rightful confirmation of divine order, Hephaestus sees outrageous tyranny. At the same time, there appears to be no way for Kratos to elicit anything beyond fearful obedience. Personifying Zeus’ *proven* superiority, Kratos lacks the material persistence of territorial possessions, symbolic titles, military assets, and monetary reserves. Whereas Bia actualises her divinity through forceful action, Kratos’ power abides only within the moment of prevailing—which is possibly why he seems compelled to repeatedly taunt Prometheus, despite his cleverness, proved unable to outwit Zeus (lines 59, 61, 87). Perhaps it is consciousness of his own ephemerality that informs Kratos’ reluctance to exit the scene with Bia and Hephaestus, so that he can remain as long as possible with the prisoner and bask in the glory of his defeat (lines 79-87).

 Kratos *needs* to have the last word, irrespective of whether it makes him appear petty or spiteful. There seems little else he can contribute to ensuring Prometheus’ imprisonment will endure, or that it will be recognised as just. Indeed, Kratos’ loyalty to Zeus is based purely on there being no other deity who has proved themselves superior (Prometheus’ premonition, that one of Zeus’ own offspring is fated to bring about his overthrow, is later used as leverage when appealing his punishment; lines 908-35). Despite Hephaestus’ assurances that Prometheus’ arms “have been [permanently] fastened,” Kratos continues issuing imperatives, acutely aware that Prometheus remains “wondrously clever at finding ways out of impossible situations” (line 59). Kratos’ supremacy thus entails multiple dependencies: not only Zeus’ divine sanction, but the supportive essences of his divine siblings Bia (Force), Nike (Victory), and Zelus (Rivalry). ^19^ These divine siblings in turn depend upon humanity’s recognition of their authority, explicitly invoked in the lamentations of the Chorus (who sympathise with Prometheus’ rebellion), and through the spectatorship of Aeschylus’ audience (lines 128-50). For his part, Prometheus remains shackled but hardly seems *dominated*. In the face of kratic prevailing, he still harbours hope for a future reversal of fortune. Thus, the domineering character of *kratos* contains within it an ever-present possibility of reversal—it is within the sportive flux, not the final result that Kratos resides.

 For contemporary manifestations of *kratos* we might be tempted to look to mass mobilisations outside established processes, whose bonds of solidarity wax and wane, and must be continually reforged. Such exercises are rarely described as ‘constructive’ (in terms of offering new proposals or fashioning new policy instruments) but rather ‘obstructive,’ insofar as they demand accountability but also serve to frustrate governmental initiatives. Media coverage of protests typically consist of interviews with strikingly coiffured, red-faced marchers, shouting their message over the din of the crowd. Fearful reports of neo-Nazi infiltrators and ‘black bloc’ agitators abound whenever people take to the streets. These and other discordances are used to discredit collective action as an effective political tool. ^20^

 Certainly the amorphousness of mass agency makes it harder to identify direct lines of accountability or to stabilise contradictory, chaotic impulses around a central aim. But we must also not forget that what motivates spontaneous, ‘unaccountable’ collective actions is a lack of accountability and competence on the part of leaders and institutions (as the insufficient responses by the establishment to climate change, disease pandemics, police violence, and regressive legislation painfully attest ^21^).

 The *kratos* of the ordinary dēmos is primarily a reactive power which can easily turn reactionary. While such obstructionism may be seen as a reason to discount the *kratos* of the modern dēmos, to relinquish the threat of noncompliance would be to abandon any hope of ‘the people’ exerting political control, as those within the establishment themselves concede:

“If you want to pull the party—the major party that is closest to the way you’re thinking—to what you’re thinking, you must— *you must—*show them that you’re capable of not voting for them. If you don’t show them you’re capable of not voting for them, they don’t have to listen to you. I promise you that. I worked within the Democratic Party. I didn’t listen, or have to listen, to anything on the left while I was working in the Democratic Party, because the left had nowhere to go.” ^22^

Despite its unruliness, the impulses of demotic *kratos* can often be seen to coalesce around collectively virtuous aims: protesting military interventions, abuses of office, ecological catastrophes, and so on. But there is nothing inherent to *kratos* to prevent its compulsive demonstrability accelerating towards insidious ends, such as the January 6, 2021 storming of the US Capitol building, driven in part by the conspiratorial rejection of ‘normal’ political contestation and the declared results of the 2020 presidential election. ^23^ For a riotous minority of Donald Trump supporters, elections are no longer a facilitator but an obstacle to ‘winning,’ thus confirming the darkest concerns about populist demagogues. Yet such was the level of affective investment in reaffirming the previous 2016 ‘win,’ it took relatively little demagogic encouragement to steer resentment towards a symbolic target. ^24^ Among supporters of the Q-Anon conspiracy, the commitment to ‘winning’ made it impossible to acknowledge loss as real; the fantasy of a secret intelligence operative working behind the scenes to expel a Satanic cabal from deep inside the federal government easily deflected inconvenient counter-evidence. ^25^ The storming of the US Capitol was motivated by a toxic stew of incoherent half-truths; it had little strategic planning; the participants were soon expelled, tracked down, and arrested. ^26^ If a common goal could be projected onto an incoherent array of motives (Q-Anon, white nationalism, anarcho-capitalists, and fundamentalist Christians) the January 6th rioters sought to prove that it was *they*, not their representatives, who had the ‘last word’ on deciding political leadership. Nevertheless, during the very brief period in which the protestors overran halls of Congress, it was clear that few if any had a particular idea *what to do* with their ‘prize.’ ^27^

 The accelerated militancy and rapid dissolution of the ‘insurrection’ manifest some of the uglier aspects of *kratos* as I have tried to define it. With alarming ease the boastful playacting of online message boards exploded into reality. The supremacy itself proved brief and self-destructive. But what the January 6th rioters share with their Congressional targets is the expressed conviction that the greatest danger to democracy is that the ‘wrong’ people will seize control of otherwise virtuous institutions. ^28^ *Kratos* infuses political gambits with a simple and deranged logic: whoever wins is somehow ‘right’ about *something* by virtue of having *won*—not because might makes right, but for *whichever* specific reasons those identifying with the victor project onto the victory. The lessons to be drawn from the win, along with the sustainability of the winning coalition, are all tangential to the punctuated fulfilment of *kratos.* This brings us to the question of whether the thraldom of competitive victory can ever be nurtured in such a way as to facilitate a more constructive and stable politics. To address this question, we turn to Josiah Ober’s work on the political dynamics of mass and elite actors in ancient Athens, and the attempted ‘domestication’ of democratic power.

## (3) The Orator as Teacher

Given the cloistered character of academia, it is likely that Josiah Ober is one of the few classicists contemporary political theorists are acquainted with. This is in no small way a result of Ober’s conscientious efforts to bridge traditional disciplinary divides. Nevertheless, while Ober remains an indispensable entry point for understanding contemporary democracies in light of the problems confronting ancient polities, it is equally important to avoid uncritically adopting his conclusions. “The Original Meaning of Democracy” was originally published in *Constellations*, a journal generally devoted to the Frankfurt School tradition of critical social theory, which hopefully augurs well for future cross-pollination between disciplines. Here he offers an alternative reading of democratic power that de-emphasises its domineering character:

Demokratia is not just “the power of the dēmos” in the sense “the superior or monopolistic power of the dēmos relative to other potential power-holders in the state.” Rather it means, more capaciously, “the empowered dēmos”—it is the regime in which the dēmos gains a collective capacity to effect change in the public realm. And so it is not just a matter of control of a public realm but the collective strength and ability to act within that realm and, indeed, to reconstitute the public realm through action. ^29^

Up to a point, I have followed Ober’s basic approach to clarifying the problems facing democracy by attempting to recover some of its ‘original meaning’ and attending to the way power is channelled through the political system. For Ober, a well-functioning democratic regime is generated and reproduced through “a socially diverse body of individuals, each capable of choosing freely in his own interests.” ^30^ From here, I think it is worth pursuing how our interpretations differ. Despite foregrounding the exercise of power by the masses, Ober has a tendency to grant outsized influence to elite orators, whose pedagogic steering facilitates the “collective capacity to effect change.” In earlier work, Ober explicitly describes democracy as an educative regime, with a unique capacity to elevate the cooperative agency of citizens:

Athens was a democracy, not just because the ordinary citizen had a vote, but because he was a participant in maintaining the political culture and a value system that constituted him the political equal of his elite neighbour. Through publicly performed speech acts, democratic institutions were implicated in an ongoing process of defining and redefining the truths used in political decision making and of assimilating local knowledges into an overarching democratic knowledge. ^31^

Ober smooths the jagged edges of contestation, so that political aims and actions gradually harmonise around monad-like repositories of democratic ‘knowledge.’ The relative success of democracy is then measured by the legibility of its foundational norms and the willingness of subjects to adopt (and occasionally expand upon) those principles. Such a model grants a clear catalytic role to the orator in shaping opinion and assimilating local knowledges. What is less clear is how the participation of the general citizenry extends beyond attentive spectatorship—and in both early and later works Ober offers little evidence to support his claim Athenian citizens conceptualised their political agency in this way. In the 2008 essay, Ober makes several rapid connections: from *kratos* to *isokratia* (an uncommon expression for ‘equality,’ found in Herodotus during the debate against restoring ‘tyranny’ to Athens ^32^), to *isonomia* (a more prevalent term, conveying ‘equal [application] of the law’), to *isegoria* (‘equal [public] address’) granted via participation in the Assembly, and then back to *isokratia.* It is by virtue of this loose etymological link to the *iso-* prefix, that *kratos* can become an unproblematic ‘good’ shared in common. ^33^ Ober ends the essay with a quote from another fourth century orator, Demosthenes, in support of his contention that the legitimating power of democracy stems from the “relationship between law, action, and the public good”:

[21.225] [T]he laws are powerful [ *ischuroi*] through you and you through the laws.
You must therefore stand up for them in just the same way as any individual would stand up for himself if attacked; you must take the view that offences against the law are public concerns [ *koina nomizein*].

To provide context, the above passage is taken from one of Demosthenes’ most famous courtroom indictments, in which he accuses his bitter political rival, Meidias, of ‘impious outrage’ (hybris). ^34^ The origins of their dispute date to 348 BCE, when Demosthenes, having been appointed khoregos (the public religious official overseeing theatrical productions for the annual Dionysia festival) was allegedly assaulted by Meidias in full view of attending spectators. The attack was the culmination of an extensive campaign of harassment and sabotage by Meidias, who was presumably intent on preventing Demosthenes from receiving a coveted drama prize. In an earlier and extended discussion of the same case, Ober presents Demosthenes’ rhetorical strategy as an attempt to frame Meidias’ behaviour as endangering civic peace. ^35^ Knowing his audience was likely composed of citizens with little sympathy for elite rivalries, Demosthenes brandishes Meidias’ hubristic contempt for norms posed a threat to all citizens, especially those who did not share his wealth and privilege. However, Ober’s translation cuts Demosthenes off mid-sentence, neglecting this final rhetorical flourish:

[21.225]…you must consider that you share in the wrongs done to the laws, by whomsoever they are found to be committed; and no excuse—neither public services [ *leitourgías*], nor pity [ *mét’ éleon*], nor personal influence [ *métʼ ándra medéna*], nor forensic skill [ *méte téchnin*], *nor* anything else *must be devised* [ *medemían heuristhai*] whereby anyone who has transgressed the laws shall escape punishment. ^36^

In Ober’s truncated version, we get the impression that Demosthenes is high-mindedly invoking rule of law as a public good. But when we view the original passage, the stakes are presented quite differently, with Demosthenes’ repeated, negative inducements ( *méte*—‘neither,’ ‘nor’) provoking his audience to block all avenues by which Meidias might escape conviction. Contrary to the virtuous pedagogy Ober projects, the orator seems more interested in the unseemly, prosecutorial impulse of *kratos*. Not only must the jury condemn the wrongness of Meidias’ actions, they must *exercise* juridical power, and to echo the now-familiar refrain, lock him up. 

 Attending to Demosthenes’ language in the above passage also reveals how Ober ‘recovers’ the original meaning of dēmokratia by quietly substituting kratos with a more harnessable ‘capacity,’ strength ( *ischuroi*)—effectively sublimating the unmediated exercise of democratic power into a “capacity of a public to make good things happen in the public realm.” ^37^ Although he does not identify any conceptual or etymological link between the two terms, Ober effectively transmutes the performative exercise of power into the tacit endorsement of the rule of law. But this fails to contend with Demosthenes’ strategy of turning a personal insult into a socially destabilising hubris, requiring the full force of the democratic polis to be mobilised. Demosthenes needs to be *seen* to win over Meidias, and so makes common cause with a dēmos that is already invested with a need to defend their ‘supremacy’:

[21.220] [W]henever a solitary victim fails to obtain redress, then each one of you must expect to be the next victim himself, and must not be indifferent to such incidents nor wait for them to come his way, but must rather guard against them as long beforehand as possible.

Ober’s reading reduces the agency of the dēmos to a ruminative spectatorship, punctuated occasionally by disgruntled or supportive noises from those seated in the Assembly or dikasteria. ^38^ And while Demosthenes’ case may lightly touch upon a concern for civic norms, his rhetorical strategy is all but designed to provoke ire, and to push the jurors to triumph over corrupt elites. Both the orator and his listener are locked in the all-consuming logic of *kratos*—the demonstrated defeat of their opponent. Of course, this is all still speculative; we will never really know what Athenian citizens and jurists ‘wanted’ with their power. Still, it is possible to further interrogate Ober’s pedagogical-rhetorical reading of *kratos* in light of contemporaneous depictions of Athenian juries. Let us shift our focus then, from elite oratory to the bawdy medium of Old Attic Comedy, as I believe this will help unlock a further crucial dimension of the essential problem of *kratos* I have sought to reconstruct.

## (4) Bdelycleon’s dilemma

The action of Aristophanes’ fifth century comedy, Wasps (originally presented in competition at the Lenaea festival of 422 BCE), centres around the troubled home of a retired soldier, Philocleon, whose name evokes reverence for the boorish populist Athenian general Cleon. Now in his dotage, Philocleon has become obsessed with participating in jury service, as enables him to fulfil his patriotic duty by hunting down corrupt elites and other enemies of the polis. To his son Bdelycleon (whose name evokes his physical disgust towards the same Athenian general), the zealotry of Philocleon and his fellow jurists is a source of considerable social embarrassment and poses an obstacle to his own upward mobility.

 As the play opens, we learn Bdelycleon has confined his father to home in the hopes of curing him of his juridical ‘addiction.’ Bdelycleon likens his father’s insatiable prosecutorial fervour to a bodily disease ( *nóson*, line 650) rather than any institutional failing. To ‘cure’ his father, he must find a way to end this compulsion for demonstrative ‘victories’ over political enemies. Railing against his confinement, Philocleon gathers his fellow *dikasts*, and confronts his son with a spirited defence of the jury system. Regaling his audience with stories of convicting elites, Philocleon enjoys a fleeting sense of power that exceeds even divine Kratos. ^39^ And, despite his pretension to seek civilised discourse, ^40^ Bdelycleon soon finds himself swept up in the heat of competition:

[530] *Bdelycleon*: Now then, what kind of man will he show himself to be, if that’s what you’re telling him to do?
*Chorus (to Philocleon)*: See that you turn out to top this youngster in debate!
  For you can see that you face a great contest now, where everything’s at stake!

Worth noting is the way Philocleon explicitly invokes kratos towards the end of his reverie—not with respect to his past accomplishments as a soldier, but rather his immediate argumentative ‘supremacy’ over his learned son:

[635] *Philocleon*: He just thought he’d be ‘picking unwatched vines’ and getting off easy that way. He knew very well that I’m the boss in this business! [ *egò taútē krátistós eimi*]

As always, Philocleon’s kratic victory is fleeting. Bdelycleon immediately launches a counterargument (lines 650-710), revealing to his father and the other *dikasts* that they unwittingly serve the ambitions of their hero Cleon, and all for a paltry three obol salary (line 680). Bdelycleon insinuates their ‘jurophilia’ is simply a continuation of their unthinking soldierly loyalty to the Athenian empire (lines 675-80). Then as now, the wasps’ patriotism is poorly compensated, leaving them humiliatingly dependent upon their children. Worst of all, the public trial system is itself be rigged against meaningful convictions due to multifarious side-dealings between defenders and prosecutors (line 695). The power Philocleon *thinks* he experiences as a juror is at best aspirational (becoming ‘Zeus-like’ in the eyes of petitioners) since his prosecutorial powers simply follow the will of Cleon. Philocleon’s momentary flashes of juridical *kratos* may feel real, but they are empty, possibly even illusory; the needful repetition of his court service only underscores the futility of the convictions themselves.

 Having punctured his father’s inflated self-regard, Bdelycleon pushes his advantage, entreating Philocleon to abandon jury-service altogether and accept the substitute of a mock trial, which he stages in the family kitchen using household objects and pets as witnesses and defendants (lines 800-1000). Bdelycleon’s strategy resembles Ober’s Demosthenes in his hope to *transform* his father’s prosecutorial fervour through the edifying introduction of legal reasoning and civic sensibility. However, it quickly transpires that Bdelycleon’s hopes are misplaced, as Philocleon continues to insist all defendants are guilty and deserve punishment. Presented with compelling evidence that the family dog, Labes is ‘innocent’ of the charge of pilfering cheese, Philocleon refuses to yield. Eventually, the only way Bdelycleon can ensure his father arrives at the ‘correct’ judgment of acquittal is by manipulating evidence, ventriloquising testimony, and finally tricking Philocleon into placing his ballot into the ‘wrong’ voting urn (lines 990-4). Having already been ‘shaken to his depths’ by his son’s revelation that courtroom verdicts are subject to backroom dealings, Philocleon is left utterly despondent in the face of Labes’ playacted acquittal. For his part, Bdelycleon uses his father’s loss to further push his advantage, insisting he submit to attending a symposium in the hopes that sympotic refinements will fully supplant political interests (lines 1207-63).

 John Zumbrunnen reads Bdelycleon’s efforts to rehabilitate his father as illustrative of the unique potential for democratic public life to harmonise competing conceptions of freedom (“rebellious disruption” versus “responsible collective action”) under the gentle rubric of comedic self-recognition. ^41^ From this perspective, Aristophanes’ plays are analogous to Demosthenes’ speeches, in that they articulate the duties and benefits of Athenian citizenship in light of emerging conflicts along class and cultural lines. Zumbrunnen views Philocleon and his fellow ‘wasps’ as embodiments of democratic citizenship’s essential paradox: the desire not to be ruled by others engenders a desire to rule others. This defensive/oppressive need cannot be assuaged through institutional processes, but only by cultivating a subtler, comedic disposition to weather the inescapable “contingency, uncertainty, imperfection, and delay” imbuing civic life. ^42^

 I am not convinced, however, that what stirs the anger of the ‘wasps’ is their lack of a ‘firm basis for self-mastery’—as opposed to a much more straightforward thwarting of their prevailing over elites, including Bdelycleon’s own ‘aristocratic’ efforts to compel them towards more elevated interests. In this, I diverge from the conventional reading of Bdelycleon as a relatable protagonist pleading for civility in democracy. The crude logic of *kratos* absorbs the son as well: it soon becomes clear that Bdelycleon is a social climber desiring to transcend his non-aristocratic family background. He does not want to educate his father for his own sake; he wants to prove himself to be his father’s superior in every way; financially, intellectually, culturally. Not only will he master the old man, he will compel his father to exaggerate old war stories, in the hopes this might impress the other guests (line 1187). Such is the transitory, transactional nature of their relationship, there can be no hope of genuine cooperation towards a common goal, only a seesawing of power between antagonists.

 No matter who ‘wins’ each interaction, it seems neither father nor son get what they ‘want,’ leaving them trapped in the endless, virtueless circle. Philocleon is too deficient in social graces and too emotionally unstable to fit the mould his son makes for him. Bdelycleon is too desperate to capitalise upon the residual social cache Philocleon enjoys as a veteran to fully commit to domesticating his father’s unruly impulses. The symposium plan fails spectacularly, as Philocleon becomes disgracefully drunk and, by the final scene, is declared a madman by the symposiasts, and stands accused of sexual assault (lines 1299-1341; 1484-90). Even after Philocleon is recaptured and forced to sober up, his very last appearance sees him bounding out of the house seeking competitors in a dancing competition (lines 1495-1500) Denied any meaningful outlet for demonstrating kratic prevailing Philocleon never loses his appetite for ‘winning.’ Bdelycleon’s attempt to wrest *kratos* away from the dēmos fails spectacularly: instead of confining this unruly energy to the interests of governance, it spills freely into the streets.

 Left alone to their juridical pursuits, was it really inevitable that Philocleon and the wasps would reduce the polis to anarchy? Possibly—but then we would have to ignore Bdelycleon’s own assertion in the debate with Philocleon that verdicts in jury trials are routinely nullified by secretive agreements between prosecutors and defendants. If reckless juridical *kratos* can be so easily annulled, what ‘danger’ did it actually pose to anyone, aside from causing Bdelycleon embarrassment? Is the problem that Philocleon’s brash, performative convictions are *illusory*, or is it that even such ephemeral triumphs are capable of inculcating a lasting desire to repeatedly test one’s *true* power?

 Even if we impute the purest pedagogical intent to Bdelycleon, his efforts to perfect Philocleon’s moral-political agency bring only anguish and confusion to the old man, hastening his eventual descent into violent animality. It seems equally likely that Bdelycleon’s moral pedagogy is an empty conceit. In any event, these two opposing kratic champions are left in mutual incomprehension and contempt. If Philocleon serves as a cautionary tale of the untamed masses, this should not lead us to ignore the subtler nastiness of Bdelycleon, a caricature of upwardly mobile youth: a short-tempered dilettante, contemptuous of his social inferiors, and covetous of elite privilege. Whenever Philocleon ‘fails’ to comport with his son’s reformist vision, the mask slips, and the son abandons all decorum, castigating his father as an ‘ignorant oaf’ (line 1183) and an irredeemable ‘pussy grabber’ ( *choiróthlips*, line 1364).

 Bdelycleon’s desire to quell his father’s ceaseless prosecutorial impulses echoes contemporary concerns about unrestrained political agency being a threat to ‘true’ democratic sociality. ^43^ Indeed, within any nominally democratic society characterised by significant disparities in wealth and status, the desire to ‘hold elites accountable’ inevitably risks demagogic incitement (hence Bdelycleon’s anxiety over his father’s adulation of Cleon: “Which is why I kept you locked up: I didn’t want these blowhards to make a chump of you”; line 720).

 The story of Bdelycleon illustrates the dilemma: his preoccupation with curbing, curing, and containing the riotous energies of the dēmos exacerbates the dangers of *kratos.* The more he insists upon Philocleon practicing the ‘right kind’ of politics, the more committed Bdelycleon becomes to winning *over* his father by any means necessary. Rather than confronting the hollowness of political ‘victory’ all critical attention becomes focused on ensuring the ‘right side’ succeeds. This derangement comes to shape the agency of both sides. Even after he submits to Bdelycleon’s lessons in etiquette, Philocleon reveals to us in an aside that he is exaggerating his obtuseness to ‘troll’ his son and regain some semblance of revenge through the young man’s upset (line 1356). 

 Today it is the ribald antagonism of Aristophanes’ characters, not the lofty pronouncements of Demosthenes, that most resembles the post-Trump political landscape. And it is the essentially deranged logic of *kratos*, not the ignorance or instability of ‘the people’ that is democracy’s most pressing problem. The question is *not* whether the dēmos is capable of self-rule; the question is whether competitive brinksmanship is our only model for legitimising rule. Politics is too real and immediate to be a ‘game’ and too important to be conducted at the behest of winners and losers. It is not a lack of coherent normative commitments, nor mindless zealotry that drives Philocleon’s kratic compulsions. It is democratic society’s inability to accommodate *both* the aspirant rule of ‘the best’ and the desire of ‘the vulgar’ to prove no one is truly their superior. Left unanswered, the kratos of the dēmos ruminates along the institutional sidelines, its prosecutorial, obstructive, intemperate force made all the more monstrous by being denied an official space in which to thwart the Promethean designs of their betters. Try as they might to translate kratos into the artefactual power of laws, institutions, and adjudicators, democratic leaders seem only to enflame a jealous, kratic refusal to comply. Philocleon represents not only the unembarrassed fury of QAnon and COVID-19 truthers, but also the righteous outrage of #MeToo and Black Lives Matter. Perhaps the most absurd aspect of demo- *kratic* conflict is the way its melodrama can remain parenthetical to ‘actual’ problems. Only through *kratos* do we see the unfathomable result of successive ‘wins’ uncoupling the ‘winner’ from the power to accomplish anything. ^44^ Just as importantly, the vituperative nature of competition between vulgar and elite control does *not* draw ever-greater numbers into the fray. Rather the majority in a democracy resemble the incidental characters of *Wasps*, who are rarely addressed by the main players, and serve mainly as bystanders and occasional victims of the feud. Despite the violent rhetoric, the combatants themselves bear little risk in the result. Philocleon and Bdelycleon still live under the same roof—just as today we find Supreme Court justices and Congressional opponents dining and socialising together, despite supposedly representing opposite ends of the ideological spectrum. ^45^ Perversely, the stakes of perpetual kratic contestation can be either of very great or little consequence; the tenor of the debate need never match the gravity of the matter being contested. This leads us to now consider the difference between *kratos* and *agonistic* contest as a fulcrum for democratic legitimacy.

## (5) Democratic Agonism

For the Bdelycleon aristocrat, the imperative is to either tame or elevate the braying masses for the sake of societal stability; through containment, hierarchy, and hortatory steering, non-experts are ushered away from direct exercises of power or encouraged to develop expertise themselves. For Philocleon democrat, the aperture for exerting political control seems forever to recede from their grasp; ‘we the people’ must therefore fanatically defend our diminished agency as consumers of culture and commodities, or find ways to at least upset the designs of the detested elite. These tensions are not reducible to balancing political ‘spontaneity’ versus ‘stability,’ rather, as we have seen, what perpetuates kratic conflict is a performative need to be seen to prevail over obstacles and opponents. Proponents of ‘agonistic democracy’ argue along similar lines. ^46^ However, their focus inevitably turns back to the character of the dēmos in an effort to understand its essential ‘political’ potential:

[T]he aim of democratic politics is to transform antagonism into agonism This requires providing channels through which collective passions will be given ways to express themselves over issues which, while allowing enough possibility for identification, will not construct the opponent as an enemy but as an adversary. An important difference with the model of ‘deliberative democracy’ is that for ‘agonistic pluralism,’ the prime task of democratic politics is not to eliminate passions from the sphere of the public, in order to render a rational consensus possible, but to mobilise those passions towards democratic designs. ^47^

Although I would suggest that *kratos* arises from the same, essentially ‘political’ dimension of antagonism underlying ordinary politics, ^48^ what distinguishes kratic power is its subversion of any attempted domestication or imposition of collective learning. Returning to the instructive template of *Wasps*, if Ober’s dialectic of ‘mass and elite’ resembles Bdelycleon’s efforts to cultivate sympotic manners in his father, then Mouffe’s ‘conflictual consensus’ resembles the playacted trial of the family dog. In both cases, unseemly antagonisms are to be sublimated by providing space for ‘legitimate’ ideational contests and cultivating respect between partisans. ^49^ From my own reading of *kratos*, however, I have suggested that manifested moment of prevailing rarely accommodates such secondary effects. Thwarting Prometheus’ unsanctioned gift of divine knowledge; hounding a corrupt politician from office—such ‘victories’ do not serve to refine our judgment nor improve procedures for handling disputes. What ‘matters’ for *kratos* is the winning, nothing else.

 Turning briefly to Aristotle, we find in the *Nicomachean Ethics* a further, troubling thought concerning the power exerted by *kratos* through *enkrateia*, or ‘self-restraint’:

[1146a.10] But a self-restrained man must necessarily have strong and evil desires; since if a man’s desires are good, the disposition that prevents him from obeying them will be evil, and so self-restraint [ *enkrateia*] will not always be good; while if his desires are weak and not evil, there is nothing to be proud of in resisting them; nor is it anything remarkable if they are evil and weak. ^50^

Whether one’s struggle is against internal compulsions or external obstacles, in harnessing *kratos* it is never enough to simply vanquish one’s opponent. Just as ‘restraint’ is never good in itself, so any victory without significant opposition remains unremarkable. Howsoever grave or trivial the battle, kratic struggle must always convey the *persistent* and *evil* nature of the opponent to hold any merit.

 Another reason for resisting a purely ‘linguistified’ rendering of *kratos*, even within the enthusiastically oratorical culture of ancient Athens, is that it misconstrues the often non-deliberative nature of ‘mass’ agency and Athenian decisional processes in particular. As far as exercises of fourth- and fifth-century democratic *kratos* are concerned, we can be relatively certain that ‘deliberation’ in the *ekklasia* and popular courts was conducted internally; voters and jurors ‘made up their minds’ about an issue (e.g. to vote against a proposed law, or in favour of imposing *atimia* or ostracism) for which they held ‘final’ decisional authority, yet they were not expected to explain their judgments or debate amongst themselves. ^51^ Contrary to the agonistic conception of being seen to ‘take a stand,’ the institutionalised processes the dēmos held the polity firmly ‘in its grip’ preserved the anonymity of individuals, both in decisions reached through a ‘show of hands’ ( *cheirotonia*) and through the casting of potsherds ( *psephophoria*). ^52^

 There is a lingering sense of Bdelycleon’s paternalism in the agonistic model of forging ‘chains of equivalence’ to harmonise the discordant demands of collectives, thereby steering them towards shared objectives. Regardless of how loosely federated such ‘hegemonies’ are expected to be, the motivating impulse remains that of defusing the danger of latter-day Philocleons, and preventing their becoming entranced by demagogic proposals that run counter to their ‘real’ interests. ^53^ Ideology critique has its uses, but we should not just assume that ‘clarifying’ ascriptions of partisan interests are exempted from the consumptive logic of *kratos*—recall Philocleon’s bewilderment at having his worldview ‘corrected’ by Bdelycleon’s critical unmasking of the dikasteria, which was itself part of his son’s struggle to silence his father and remove this embarrassing obstacle to social advancement. *Kratos* seems to add a sulphuric stench of cynicism to all its enterprises—seeding paranoia within the ranks of grassroots protests, using communicative reason for manipulative political ‘dressage.’ In my concluding thoughts for this paper, I will consider what relevance, if any, my reconstruction of ancient *kratos* may have for discussions of contemporary political discontents.

## (6) Kratos Unbound?

In this paper I have sought to describe a distinctive mode of power I identify with *kratos*, which consists in efforts to paralyse, perplex, and prevail over perceived obstacles or opponents. I have also sketched the peculiar dynamic by which enlightened thinkers and policymakers have sought to ameliorate the violent, destabilising tendencies of kratic power, as wielded by a dēmos. Whether through forceful curtailment, incentivised redirection, or moral didacticism, these attempted domestications are themselves (aristo)kratic exercises in asserting superiority over the intemperate masses. I contend that such efforts fail to learn the lessons of Bdelycleon, whose pedagogical and hortatory principles seamlessly succumbed to the same compulsion to prevail. The shared antipathy by the dēmos against elites can encompass any number of overlapping and incompatible plaints—from antisemitic conspiracy-mongering, to vulgar Marxism, to pure anarchism—but it does not follow from these incoherences that party-disciplined, piecemeal reformism is superior.

 Presently, we find across the political spectrum significant mobilisations of anti-majoritarian sentiment. Particularly in the US, there have a been a series of devastating judicial decisions to suppress voting rights and the reproductive rights of women. ^54^ In both ‘blue’ and ‘red’ US states, there has been continued effort to circumscribe the legality of protest and unionisation. ^55^ Populist candidates may position themselves as ‘outsiders’ against elites, but when faced with the endless see-sawing of wins and losses, the eventual interest to secure lasting rule leads either to working with ‘insiders’ to gerrymander districts and revise voter registration, or retreating deeper into meta-political ideology critique ^56^ and conspiracy theorising. ^57^ ‘Winning’ is an empty, ephemeral thing—its sheer facticity grants legitimacy to the victor by convention rather than by anything inherent to contestation. In this, I am guided by Aristotle’s concern in Book IV of the *Politics*:

[Book IV 1296a.25] And these considerations also show the reason why the constitutions of most states are either democratic or oligarchical; owing to the middle class in these states being often a small one, the classes diverging from the middle status—whichever of the two, the owners of the estates or the people, from time to time has the upper hand—conduct the government on their own lines, so that it becomes either a democracy or an oligarchy. And in addition to this, because factions occur and fights between the people and the wealthy, *whichever party happens to gain the upper hand over its opponents* [ *hopotérois an mallon sumbēi kratēsai tōn enantion*], does not establish a common or equal government, but takes the superior share in the government as a prize of victory. ^58^

As Aristotle’s language makes clear, the wholly contingent nature of kratic prevailing fuels the vituperative character of the resultant prize. Aristotle also hypothesises that the potential destructiveness of *kratos* might be meliorated by reducing inequality and lowering the stakes of competition—and offers the example of a predominantly agricultural population with a baseline quality of life as affording a more benign disengagement from politics. ^59^

 Of course, we would not pretend a slave-owning, agrarian peace is feasible or desirable for silencing the noise of modern *kratos*, especially as we confront the unprecedented, cascading ecological crisis that threatens to overshadow all other political disputes. It is an understatement to say kratic contestation appears uniquely ill-suited to addressing the challenges of the Anthropocene. But such an observation is not meant to confirm the suspicions of the Old Oligarch. ‘Democracy’ is not the true problem. We have seen that foundational democratic values like free and fair elections are themselves wholly dispensable to *kratos*; elections are as likely to be considered obstacles to ‘prevailing.’ And, as the sphere of politics continues to recede from lived experience, most citizens of democratic states lack even remote acquaintance with democracy in action. Compulsory military service is thankfully less common; less happily, many people have neither the time nor the inclination to participate in deliberative mini-publics, run for elected office, take up membership in trade unions, or join in mass protests. A democratic politics that loses its meaningfulness to citizens leaves only the contest itself, and occasional vicarious investments in electoral triumph. It the absence of outlets for unmediated political power that allows ‘winning’ to become totemic for ‘democracy.’

 It is probably true that nothing short of a forced curtailment of extractive industries and the removal of incompetent leaders is likely to ensure the survival of our species. A grassroots mobilisation of *kratos* might, at first glance, seem like a remedy. But as we reacquaint ourselves with the political vocabulary we inherited, we must inspect its flaws and question *how* the deranged logic of prevailing could ever offer a viable means for collective decision-making. Although I have not offered a clear solution for the problem of *kratos*, I believe I have at least made a case for dispelling the chimaera that ‘the People’ or ‘the Elites’ must be transformed or replaced to safeguard conditions of autonomy. To remain fixated upon the character and quality of the dēmos, and preoccupied with divining lessons from each election, is to leave ourselves at the mercy of forgotten gods.
