# Supplementary material for: What’s Wrong with Democracy? The Peculiar Power of Kratos
Source: Open Res Eur. 2022 Nov 28;1:56. Originally published 2021 May 25. [Version 2] doi: 10.12688/openreseurope.13726.2 (PMC10445922; doi:10.12688/openreseurope.13726.2)
Supplement: Peer review history

# Peer review: Reviewer response for version 2

**DOI:** 10.21956/openreseurope.16596.r30428

**Published:** 2022-12-16

**Authors:** Fredrik Sixtensson

**Affiliations:** 1 Center for Statecraft and Strategic Communication, Stockholm School of Economics, Stockholm, Sweden

The author has corrected everything that needed correcting, and improved the article in other respects, too.

Is the argument persuasive and supported by appropriate evidence?

Partly

Is the work clearly and cogently presented?

Partly

Is the topic of the essay discussed accurately in the context of the current literature?

Partly

Does the essay contribute to the cultural, historical, social understanding of the field?

Yes

Reviewer Expertise:

Greek linguistics, historical linguistics, political language, democracy, politics, constitutional typology

I confirm that I have read this submission and believe that I have an appropriate level of expertise to confirm that it is of an acceptable scientific standard.

# Peer review: Reviewer response for version 2

**DOI:** 10.21956/openreseurope.16596.r30429

**Published:** 2022-12-02

**Authors:** Vasileios Adamidis

**Affiliations:** 1 Nottingham Trent University, Nottingham, UK

Happy to approve the updated version, and I hope that my review and the sources suggested have been helpful and constructive.

Is the argument persuasive and supported by appropriate evidence?

Partly

Is the work clearly and cogently presented?

Yes

Is the topic of the essay discussed accurately in the context of the current literature?

Partly

Does the essay contribute to the cultural, historical, social understanding of the field?

Partly

Reviewer Expertise:

Classical Athenian Democracy; Attic Orators; Populism; Democratic Theory; Rule of Law.

I confirm that I have read this submission and believe that I have an appropriate level of expertise to confirm that it is of an acceptable scientific standard.

# Response

**Published:** 2022-12-02

**Authors:** John McGuire

**Affiliations:** 22032, Ireland

I am very grateful to Prof Adamidis for highlighting the need to distinguish between populism and 'kratos' in the essay. His suggested references were highly pertinent and useful!

# Peer review: Reviewer response for version 1

**DOI:** 10.21956/openreseurope.14801.r29627

**Published:** 2022-07-01

**Authors:** Fredrik Sixtensson

**Affiliations:** 1 Center for Statecraft and Strategic Communication, Stockholm School of Economics, Stockholm, Sweden

This article sets out to analyse the Greek concept of *kratos*, a ‘peculiar mode of power’ familiar to the Greeks, which, as it were, has been forgotten. The author argues that if we can recapture the Greek understanding of *kratos*, which is performative and unmediated in its nature, we would be in a better position to understand spontaneous, extra-parliamentary, ‘populist’ expressions of popular power; we would begin, is the author’s hope, to consider popular discontent as something legitimate and healthy, not something that ought to be domesticated.

 The article is well-written (save for some formatting errors) and the reading of ‘ *kratic *power’ is stimulating.

 Below follows what in my view should be clarified. I will begin with some general remarks (sometimes, however, illustrating a general point by going into minute detail). Then I will proceed to details. Some points of criticisms are trivial; others are more serious and ought to be amended.

**General remarks:**

**1.** The employment of ancient sources to produce political insights is commendable. But there are problems associated therewith: it is often unclear if the author is explicating the Greek understanding of a concept or if he is extending this concept to employ it in general (and ahistorical) philosophical criticism. Both things can of course be done within the same article, especially in an article with this aim. But it must be clear when one is doing which.

 An example of this problem can be found at page eleven, where the author charges the ‘Old Oligarch’ with misunderstanding *enkrateia*:

 "What the unknown fifth-century writer (the "Old Oligarch") fails to acknowledge is that even an undisciplined *kratos* wielded by ignorant masses still constitutes true *enkrateia*, insofar as they are demonstrably able to "restrain" the actions of contemptible elites."

 The author speaks of ‘true *enkrateia*’. Does this refer to the how most Greeks interpreted this concept—so that the Old Oligarch could be refuted with reference to, say, how *enkrateia* is used by some other Greek author—or does it refer to what the author himself sees as true *enkrateia*, despite what the Greeks may have thought? The author has not provided his own definition, but I can only conclude that he has himself extended the concept; for whenever *enkrateia* bears on politics (more on this in my next point of criticism), it is precisely as something that characterizes the elite.

 It is evident that the Old Oligarch (and Aristotle) refer to a character trait, a quality of single individuals: he says that aristocrats tend to have more self-restraint than the masses. This is *enkrateia’*s common meaning. When the author is saying that undisciplined *kratos* constitutes true *enkrateia*, it comes close to saying that lack of self-restraint is self-restraint. I could understand if, on a metaphorical level, the suppression of elites would constitute a collective self-restraint, provided the demos is defined as the total citizenry. But then we use *enkrateia *to describe a sociological phenomenon with no connection to individual self-restraint, which is what *enkrateia* usually refers to. It should here be noted that, in Greek philosophy, whenever *enkrateia* (and loss thereof) is connected to politics, it is always conceived of as something pertaining to the character or soul of each individual citizen. To say that the Old Oligarch does not understand true *enkrateia* while redefining its scope in this fashion is a category error.

 But let us, arguendo, bracket these points. Then I have another point of criticism. Since *enkrateia* is about resisting certain destructive urges and temptations within the soul one should ideally be able to conceptualize the actions of the elite as precisely ‘urges and temptations’, to which the demos may succumb because it lacks moral fibre, for the metaphor to work. This argument is difficult to make, unless we think of demagogues who aimed for tyranny. But the elite tended not to approve of tyranny: they despised it.

 I take the basic argument in this passage to be that the unruliness of the masses does not contradict *kratos*. To strengthen this argument, the author could reference the common connection between *kratos* and *lack of self-restraint*, which is a staple in Homer, who sometimes uses the expression βίῃ καὶ κάρτει εἴκων ‘yielding to *bie *and *kratos*’ when someone cannot quite control their ‘will to power’, as it were. I discuss this in Sixtensson (2021, 114–117) ^5^. On this basis, it could be argued that the demos’ lack of restraint is in fact a manifestation of their *kratos*.

 The criticism about clearly defining concepts also applies to the author's use of 'democracy' and 'populism', but these are easily corrected.

**2.** The author sometimes risks misrepresenting the arguments of modern scholars, especially in his treatment of Josiah Ober’s work. Quoting is also at times inexact (but I think this is easily corrected). I will come back to this below in the detailed treatment.

**3.** The article could also benefit from more references to secondary literature concerning the meaning of certain terms to substantiate some interpretations. The references to works on *kratos* mostly consist of political theory, it seems to me; these could be complemented with philological works. Some articles that could be of benefit on specifically *kratos *are Breuil (1989) ^1^ and Breuil (1995) ^2^. When the author, in his criticism, speaks of the meaning of *ischus*/ *ischuroi *and how it differs from that of *kratos* **(p. 7), there are no references to secondary literature, not even a dictionary. I would recommend turning to Schmidt (1879, 656–697) ^4^, which compares words that pertain to strength, among them being both *kratos *and *ischus* (pp. 660–662 deal specifically with *ischus *and cognates *; *pp. 667–675 treat *kratos *and cognate). There is a useful table comparing the terms on pp. 696–697. The discussion of speech and *Kratos* in *Prometheus Bound *(which is very interesting) could perhaps be amplified from a reference to Walter (1964, 350) ^6^.

**4.** The transliteration is inconsistent. The original Greek is now reproduced with Greek, now with Roman letters. *Demos* is sometimes *d* *ē* *mos*. *Kratos* is sometimes in italics, sometimes not. Greek eta is sometimes rendered as *e*, sometimes as *ê* (and, in one passage, as *i*). 

 Now, I will turn to some details. 

**Details**

**Pp. 3-4:** " *Kratos*, as I will define it, expresses a distinctively “performative” mode of power manifesting in moments of prevailing, which encompasses winning an argument, convicting an abuser, turning the tide of battle, reversing a policy decision, conducting a mass boycott, organising a strike, blockading [p. 4] access to disputed territory, or hounding a corrupt leader from office."

 Here, it must be clarified which relation this definition bears to the Greek use. The point that *kratos* is a power "manifesting in moments of prevailing" is interesting (probably true) and could be underlined with reference to its sometimes translation ‘victory’, which is not mentioned. Perhaps my discussion in Sixtensson (2021, 109–114) ^5^ about *kratos'*s relation to victory could be of some benefit.

**P. 4:** "This also makes *kratic* power far less stable than that which is established through the rule of law or political office."

 I am not sure about this statement. If we are to trust the argument of Thrasymachus in the *Republic *(338), all institutional rule is in fact underpinned by *kratos*. From this would follow that if *kratos* were to disappear, then power exercised through institutions would also disappear. The question then is, of course, if the author is talking about Greek *kratos* or his own definition (which should be made explicit).

**P. 4:** "As for “democracy,” the paradoxical notion of supreme power exercised by unexceptional masses has led theorists like Jacques Rancière and Sheldon Wolin to present the phenomenon as essentially undefinable, a-constitutional, and anarchic."

 Plato, too, suggests that democracy is anarchic, in *Republic* 562de. I simply point this out in case the author thinks it appropriate to invoke him as well.

**P. 4:** "The manhandling is left to Bia, who silently obeys her brother’s instructions—pinning down Prometheus’ arm, then the other, then his chest and legs—all the while ignoring Hephaestus’ pleas for clemency."

 Since we lack stage directions, it is not clear what Bia does. It is admittedly a fair assumption that Bia is the one manhandling Prometheus, but it is by no means certain; it could be that Hephaestus is the only one who touches Prometheus. This should be made clear, as I think it may affect the argument. Must *bia* be exercised for *kratos *to be active, or is the looming threat of *bia* enough? (I would argue the latter.)

**P.4:** "In response, Kratos reminds Hephaestus they are both subject to the exacting standards of a “tough appraiser,” and must do their utmost to ensure Prometheus comes “to accept the tyranny of Zeus” (line 10)."

 The Greek says that "the appraiser of the works is tough", without saying whose works; that is, Kratos does not explicitly say that they both are subject to these standards. Not including Kratos makes sense, as Kratos is the embodiment of (Zeus’s) power, not precisely its subject.

 The line about the tough appraiser is line 77, i.e., almost seventy lines apart from the statement that Prometheus must accept the tyranny of Zeus; the passage should be rephrased to reflect this.

**P. 5:** "These divine forces in turn depend upon the public’s recognition of their authority…"

 This claim needs to be specified, given that Bia is included among the divine forces. Bia is compulsion and violence. Is it appropriate to talk about ‘recognition’ apropos compliance following from violence?

**P. 5:** "Aristotle’s description of democracy in the *Politics* as a regime-type born of severe inequality"

 The quoted passage does not refer to democracy in general, but to the most extreme form thereof. This should be specified.

**P. 6:** "Yet, despite foregrounding the exercise of power by the masses, Ober grants an outsized role to *elite orators*, whose mediative influence steers the "collective capacity [of the dēmos] to effect change.""

 The two block quotes illustrating these contradictory tendencies are fourteen years apart. The ‘mediative influence’ of the orators is from the article from 1994, but it is quoted as if it bears directly on what Ober says about ‘the collective capacity to effect change’, which is a quote from the 2008 article. There is a risk of straw-man argumentation. It should be made clear that Ober’s views are constant enough that this is proper.

**P. 6:** "Ober ends the essay with a quote from the fourth century orator, Demosthenes, which supports his contention that the legitimating power of democracy stems from the "relationship

 between law, action, and the public good": [21.225] [T]he laws are powerful [ischuroi] through

 you and you through the laws. You must therefore stand up for them in just the same way as any individual would stand up for himself if attacked; you must take the view that offences against the law are public concerns [koina nomizein]."

 There is no footnote indicating page number in Ober's essay, nor a footnote indicating where the quote about the "relationship between law, action, and the public good" comes from. It turns out that this is from the end of the essay 'The original meaning of "democracy', but without indicating this in a footnote, it seems as if it is coming from the later text. 

**P. 7:** "[21.225]…you must consider that you share in the wrongs done to the laws, by whomsoever they are found to be committed; and no excuse—neither public services [ *méte litourgías*], nor pity [ *méte éleon*], nor personal influence [ *métʼ ándra midéna méte téchnin*], nor forensic skill [ *méte heuristhai*], nor anything else—must be devised whereby anyone who has transgressed the laws shall escape punishment."

 The quote is not accurate: the Greek for ‘nor forensic skill’ in the original is μήτε τέχνην μηδεμίαν εὑρῆσθαι. In the author’s quote, " *méte téchnin"* is mistakenly assigned to ‘personal influence’ and the ‘ *medemían*’ before ‘ *heuresthai*’ has become ‘ *méte*’. Besides, Greek *eta* is, inconsistently with the rest of the article, reproduced as Latin ‘I’ in *litourgias*, *midena*, *technin*, *heuristhai*.

**P. 7:** "Contrast this to Ober’s euphemistic framing of democratic agency, in which the orator establishes “the limits of behaviour appropriate to the most powerful individuals in Athenian society [and] the public consequences of allowing those limits to be breached.""

 I do not have access to *The Athenian Revolution*, but have found Ober (2004), published in another volume ^3^. If the article is the same, then this is appears to me to slightly misrepresent what Ober is saying. Ober does not say that the ‘orator establishes’ limits of behaviour: he says that Demosthenes’s speech is ‘openly concerned’ with establishing such limits. This is a claim about Demosthenes’s goals, whereas the author’s wording suggests that Ober thinks he succeeds in doing this, and that this was something that orators could do (and normally did). Since the author is talking about democratic agency, the difference is important.

**P.7:** "Attending to Demosthenes’ language in the above passage also reveals how Ober, in establishing the “original meaning” of *dēmokratia*, quietly substitutes the unwieldy drive of *kratos* with the more harnessable capacity of strength ( *ischuroi*), effectively transforming democratic “power” into a virtuous “capacity of a public to make good things happen in the public realm.” Although he does not identify any conceptual or etymological link between the two terms, in transmuting the performative exercise of power into the tacit endorsement of the rule of law, Ober makes the hortatory steering of elite orators an indispensable catalyst for democratic systems.

 Here, a reference to Schmidt (1879, 656–697) ^4^ could help the author's argument. (I must also confess that given the lack of footnote reference mentioned above, I did not realize that the passage about *ischuroi *was from the 'The original meaning of "demokratia"' text. Having realized this, I think the author is onto something about the move Ober makes here).

**P.7:** "let us consider a different courtroom speech in which Demosthenes actually invokes *kratos*:"

 In the quoted passage, Demosthenes uses the phrase ‘ *kata kratos*’. This is a fixed expression that can sometimes mean ‘by force’, but often has the bleached meaning ‘totally’. These fixed expressions are lexemes in their own right and should be treated with some caution. It is a bit like saying that the expression ‘run like hell’ invokes Hell.

 I also think that the difference between *ischus *and *kratos* that the author sees in Demosthenes's speeches should be clarified even further. I am not sure if I understand fully, but I suspect that clarifying it would be easy. 

**P. 11:** What the unknown fifth-century writer (the ‘Old Oligarch’) fails to acknowledge is that even an undisciplined *kratos* wielded by ignorant masses still constitutes true *enkrateia*, insofar as they are demonstrably able to ‘restrain’ the actions of contemptible elites.

 I have dealt with this at length above.

Is the argument persuasive and supported by appropriate evidence?

Partly

Is the work clearly and cogently presented?

Partly

Is the topic of the essay discussed accurately in the context of the current literature?

Partly

Does the essay contribute to the cultural, historical, social understanding of the field?

Yes

Reviewer Expertise:

Greek linguistics, historical linguistics, political language, democracy, politics, constitutional typology

I confirm that I have read this submission and believe that I have an appropriate level of expertise to confirm that it is of an acceptable scientific standard, however I have significant reservations, as outlined above.

## References

[1] : Κράτος et sa famille chez Homère. Étude sémantique, In: M. Casevitz (ed.), Études homériques. *Travaux de la Maison de l'Orient 17. Lyon: Maison de l'Orient Méditerranéen* .1989;17-53

[2] : De κράτος à δημοκρατίη : Une famille des mots chez Hérodote. *Ktèma 20* .1995;71-84

[3] : Power and Oratory in Democratic Athens: Demosthenes 21, Against Meidias, In: EW Robinson (ed.), Ancient Democracy: Readings and Sources. *Malden, MA: Blackwell* .2004;232-247

[4] : Synonymik der griechischen Sprache. Vol. 3. *Leipzig: Teubner* .1879;

[5] : Dēmokratia: The Prehistory of a Word in Relation to the Greek Typology of Constitutions. *Uppsala University* .2021; Reference source

[6] : Power and Violence. *American Political Science Review* .1964;58(2) : 10.2307/1952867 350-360 10.2307/1952867

# Peer review: Reviewer response for version 1

**DOI:** 10.21956/openreseurope.14801.r27616

**Published:** 2021-10-11

**Authors:** Vasileios Adamidis

**Affiliations:** 1 Nottingham Trent University, Nottingham, UK

This is a useful and noteworthy attempt to explore the meaning of *kratos *in the *demo-kratia* with particular focus on the direct democracy of classical Athens. There is also an effort to link the notion of *kratos* with contemporary manifestations of ‘exercise of power’, particularly as a reaction 'from the margins' to established and more institutionalised practices (quite often, of the ‘establishment’). This is a commendable, cross-disciplinary attempt which has the potential to make a contribution to recent scholarship on the field, facilitating a better understanding of the Athenian democracy and the philosophical conception of *kratos* as the exercise of power with unique characteristics, and also draw links with modern practices and movements.

 The article concentrates on the analysis of a few literary sources, ranging from mid-5th century to mid-4th century BCE, and discusses influential accounts on the relationship between ancient democracy and its modern conceptions (esp. Ober). More sources on the origins of democracy could have informed the discussion (e.g. Harris 2016). Links with the concept of populism are also offered, but these are sporadic. However, more detailed and systematic treatment of this matter would have provided a useful connecting point between the discussion of power relations in the ancient *demo-kratia* and the emergence of contemporary movements discussed in the text. (For detailed discussion of populism and its applicability in classical Athens, see Adamidis 2019, 2021a, forthcoming; Beigel 2017). Similarly, references to political oratory and the role of demagogues could be supplemented by the findings of more extensive studies in the field (e.g. Mann 2007).

 Further streamlining of the main argument(s) would certainly be beneficial, in addition to a more robust methodological treatment of and a more systematic approach to the selection, use and interpretation of the ancient sources.

 The attempt to approach ancient sources belonging to a range of genres (tragedy, comedy, forensic oratory, political theory) is definitely challenging, yet rewarding, therefore their more systematic treatment and an explanation of the rationale for their selection would be welcome. The extensive discussion of and reliance on *Prometheus Bound* and *The Wasps* require caution, as the works of Athenian drama should rarely, if ever, be taken at face value, and they can often be misleading. For example, although the article offers some interesting observations in the discussion of *Prometheus Bound* (e.g. *kratos* as the exercise of power through the medium of speech; the need for the public recognition of authority; loyalty to the powerful etc.) it seems to overlook that Kratos, despite its personification in the play, is still Zeus' medium for the exercise of his power and authority and the projection of his strength, not as a widely accepted legitimate sovereign but (as he is frequently referred to in the play) a *tyrant*. Therefore, the aforementioned characteristics of *kratos* may be malleable and adaptable depending on who exercises it. 

 A note on the methodology would be useful early in the article, as there appears to be an effort to engage in literary and linguistic analysis of the texts, while placing them within their appropriate historical, social, and cultural context, though without a clarification of the exact methodological approach to be followed.

 The article could further improve by a more detailed clarification of the terminology used. For example, there are (understandably) frequent references to essentially contested concepts such as 'democracy' and ‘populism’ and their interconnectedness, so a definition of them would certainly assist the reader to understand the connections between ancient and modern uses of the terms, and the links drawn between their respective manifestations and characteristics (see Adamidis 2021b and 2021c).

 Reference to 'norms related to democracy' such as equality before the law, could be more usefully associated with the 'liberal' aspect of modern, representative democracy, or with the concept of the 'rule of law', in order to offer an insight into their presence in ancient *demokratia* and their relevance to / compatibility with *kratos*. Additionally, links and comparisons between the notion of *kratos* and the concept of sovereignty, as well as a discussion of their relationship with law, would expand the scope of the enquiry and engage a wider audience (on this, see Morgan 2003; Bourke/Skinner 2016; Kalyvas 2005; Eleftheriadis 2010).

 The need for a more rigorous definition of democracy is particularly highlighted when it is claimed that “democracy *can* be achieved through the enlightened mediation of leaders and orators, who are singularly capable of smoothing out the jagged edges dividing democracy from aristocracy”. This statement seems to project a modern understanding of the term to ancient political thought.

 Discussion of the complicated relationship between *demo-kratia* and the rule of law as a concept which accompanied Athenian democracy could be usefully, though concisely, incorporated in the text, as this would be illuminative of the Athenian views on kratic power. The author argues that “kratic power abides within the provenness of authority, as opposed to tethering its legitimacy to institutional mandates or legal precedents. This also makes kratic power far less stable than that which is established through the rule of law or political office.” These statements could potentially be qualified if the demo-kratic power was grounded on the legitimacy afforded to it by its adherence to the rule of law. (See Canevaro, 2017; Harris 2013; Adamidis 2017; Gagarin 2020.) Finally, the claim that “Another reason for resisting a linguistified rendering of *kratos*—even within the ceaselessly oratorical culture of ancient Athens—is that it misconstrues the decidedly non-deliberative nature of mass agency in general, and Athenian decisional processes in particular (which remained distinct from the counsel of the orators)” could possibly be further informed, or even qualified, in light of recent studies on the field. (See, for example Cammack 2017; Canevaro 2018; Tacon 2001.)

Is the argument persuasive and supported by appropriate evidence?

Partly

Is the work clearly and cogently presented?

Yes

Is the topic of the essay discussed accurately in the context of the current literature?

Partly

Does the essay contribute to the cultural, historical, social understanding of the field?

Partly

Reviewer Expertise:

Classical Athenian Democracy; Attic Orators; Populism; Democratic Theory; Rule of Law.

I confirm that I have read this submission and believe that I have an appropriate level of expertise to confirm that it is of an acceptable scientific standard, however I have significant reservations, as outlined above.

## References

[1] : Character Evidence in the Courts of Classical Athens: Rhetoric, Relevance and the Rule of Law. *Routledge* .2017;

[2] : “Manifestations of Populism in late 5th Century Athens.” In: D. Frenkel and N. Varga (eds.), New Studies in Law and History. *ATINER* .2019;11-28

[3] : Populist Rhetorical Strategies in the Courts of Classical Athens. *ATHENS JOURNAL OF HISTORY* .2020;7(1) : 10.30958/ajhis.7-1-2 21-40 10.30958/ajhis.7-1-2

[4] : Populism and the Rule of Recognition. *Populism* .2021;4(1) : 10.1163/25888072-BJA10016 1-24 10.1163/25888072-BJA10016

[5] : Democracy, populism, and the rule of law: A reconsideration of their interconnectedness. *Politics* .2021; 10.1177/02633957211041444 10.1177/02633957211041444

[6] : Populism in Power? A reconsideration of the Athenian Democracy of the Late 5th century BC (forthcoming). *Journal of Ancient Civilizations* .

[7] : “Das klassische Athen. Geführte oder verführte Demokratie?” In: G. Eckert and T. Beigel (eds.), Populismus. Varianten von Volksherrschaft in Geschichte und Gegenwart. *Aschendorff Verlag* .2017;39-54

[8] : Popular Sovereignty in Historical Perspective. *Cambridge University Press* .2016;

[9] : Deliberation in Classical Athens: Not Talking, but Thinking (and Voting). *SSRN Electronic Journal* .2012; 10.2139/ssrn.2161074 10.2139/ssrn.2161074

[10] : The Rule of Law as the Measure of Political Legitimacy in the Greek City States. *Hague Journal on the Rule of Law* .2017;9(2) : 10.1007/s40803-017-0054-1 211-236 10.1007/s40803-017-0054-1

[11] : “Majority Rule vs. Consensus: The Practice of Democratic Deliberation in the Greek Poleis.” In M. Canevaro et al (eds.), Ancient Greek History and Contemporary Social Science. *Edinburgh University Press* .2018;101-156

[12] : Law And Sovereignty. *Law and Philosophy* .2010;29(5) : 10.1007/s10982-010-9077-7 535-569 10.1007/s10982-010-9077-7

[13] : Democratic Law in Classical Athens. *University of Texas Press* .2020;

[14] : The Rule of Law in Action in Democratic Athens. *Oxford University of Oxford Press* .2013;

[15] : “The Flawed Origins of Athenian Democracy.” in A. Havlíček, C. Horn and J. Jinek (eds.), Nous, Polis, Nomos. Festschrift Francisco L. Lisi. Studies on Ancient Moral and Political Philosophy. *Academia Verlag* .2016;43-55

[16] : The Popular Constituent Sovereign and the Pure Theory of Democratic Legitimacy.2008; 10.1017/CBO9780511755842.007 88-126 10.1017/CBO9780511755842.007

[17] : Demos (a)kurios? Agenda power and democratic control in ancient Greece. *European Journal of Political Theory* .2021; 10.1177/14748851211015331 10.1177/14748851211015331

[18] : . Die Demagogen und das Volk: Zur politischen Kommunikation im Athen des 5. Jh. v. Chr. Klio Beihefte N.F. 13. *De Gruyter* .2007;

[19] : Popular Tyranny. Sovereignty and its Discontents in Ancient Greece. *University of Austin Press* .2003;

[20] : EcclesiasticThorubos : Interventions, Interruptions, and Popular Involvement in the Athenian assembly. *Greece and Rome* .2001;48(2) : 10.1093/gr/48.2.173 173-192 10.1093/gr/48.2.173
